# Symbolic transfer entropy reveals the age structure of pandemic influenza transmission from high-volume influenza-like illness data

**DOI:** 10.1098/rsif.2019.0628

**Published:** 2020-03-18

**Authors:** Stephen M. Kissler, Cécile Viboud, Bryan T. Grenfell, Julia R. Gog

**Affiliations:** 1 Department of Applied Mathematics and Theoretical Physics, University of Cambridge, Wilberforce Road, Cambridge, UK; 2 Department of Immunology and Infectious Diseases, Harvard T.H. Chan School of Public Health, Boston, MA, USA; 3 Fogarty International Center, National Institutes of Health, Bethesda, MA, USA; 4 Department of Ecology and Evolutionary Biology, University of Princeton, Princeton, NJ, USA

**Keywords:** symbolic transfer entropy, pandemic influenza, age structure, electronic medical records, influenza-like illness

## Abstract

Existing methods to infer the relative roles of age groups in epidemic transmission can normally only accommodate a few age classes, and/or require data that are highly specific for the disease being studied. Here, symbolic transfer entropy (STE), a measure developed to identify asymmetric transfer of information between stochastic processes, is presented as a way to reveal asymmetric transmission patterns between age groups in an epidemic. STE provides a ranking of which age groups may dominate transmission, rather than a reconstruction of the explicit between-age-group transmission matrix. Using simulations, we establish that STE can identify which age groups dominate transmission even when there are differences in reporting rates between age groups and even if the data are noisy. Then, the pairwise STE is calculated between time series of influenza-like illness for 12 age groups in 884 US cities during the autumn of 2009. Elevated STE from 5 to 19 year-olds indicates that school-aged children were likely the most important transmitters of infection during the autumn wave of the 2009 pandemic in the USA. The results may be partially confounded by higher rates of physician-seeking behaviour in children compared to adults, but it is unlikely that differences in reporting rates can explain the observed differences in STE.

## 1. Introduction

Age is a key predictor of a person’s rate of both acquiring [1–6] and transmitting [1,7,8] influenza. Children tend to contribute more to influenza transmission than adults do [1,4,7], but the precise epidemiological roles of different age groups can shift from season to season [9] and may change markedly in pandemic years [10]. From a public health perspective, untangling the relative roles of different age groups could help guide targeted vaccination strategies [7,11–13] and other age-related interventions, like the selective closure of schools [14–16]. However, data with sufficient resolution to identify detailed epidemiological relationships between age groups have so far been scarce, and even when such data exist, current methods are insufficient for reliably uncovering those relationships.

Electronic medical records (EMRs) help address the issue of data scarcity by providing high-volume influenza-like illness (ILI) incidence data with detailed age structure [17]. EMRs are routinely produced by physicians for insurance purposes during the majority of outpatient visits in the USA [17]. Since EMRs generally contain syndromic illness classifications, EMR-based estimates of influenza incidence are subject to noise from non-ILI respiratory infection. EMR-based disease incidence estimates are also subject to geographical and demographic variation in physician-seeking behaviour. Laboratory-confirmed influenza cases, as collected routinely by the Centers for Disease Control and Prevention (CDC) [18], provide more specific estimates of influenza incidence, but at substantially lower volume. Influenza incidence estimates from online search platforms and social media websites like Google [19] and Twitter [20] can provide massive amounts of data, but the reliability of these sources has been called into question, and they lack detailed age information [21]. Dedicated online platforms such as FluNearYou in the USA and FluSurvey in the UK, which gather reports of ILI symptoms from community volunteers [22,23], hold some promise for supplementing traditional ILI data streams [24–26], but represent a relatively small convenience sample of the population. So, while other data sources exist, EMRs offer a relatively promising and so-far underused source of fine-scale data on influenza incidence in the USA [17,21].

Previous attempts to infer the relative importance of different age groups for the transmission of influenza have sought to either reconstruct the explicit next-generation matrix (NGM) [3,27,28] or to infer the relative risk of infection between age groups [4]. The NGM-based methods have only been applied to scenarios with at most two age groups (children and adults), in part because they require strong assumptions about the structure of the NGM which become increasingly unrealistic as the number of age classes grows. The relative risk method [4,29,30] has been used to rank the importance of five age groups for the transmission of influenza, but requires data with high specificity for influenza, effectively precluding ILI data streams and the use of EMRs in particular. These methods are generally aimed at identifying optimal vaccine allocation strategies using data from the initial phase of an epidemic.

Symbolic transfer entropy (STE) [31] offers a way to infer the relative transmission importance of possibly many age groups from ILI data. STE is an extension of transfer entropy (TE) [32], which measures the amount of information the past states of one stochastic process provide about the transition probabilities of another. Intuitively, the TE is a measure of the amount of information ‘transferred’ from one stochastic process to another. To compute the STE, a time series is symbolized using a scheme that encodes its qualitative structure in a low-dimensional space, and then the TE is calculated from the relative frequencies of these symbols. The symbolization scheme makes the STE robust to moderate observational noise and to systematic shifts in amplitude, which in the context of EMR ILI data might arise from the presence of non-influenza ILI cases and from differences in reporting rate between age groups. These benefits come with the trade-off of requiring relatively large amounts of data compared to existing methods for inferring the age structure of disease transmission and providing only a relative ranking of transmission importance. STE has been used to study epileptogenic neural signals and the dissemination of information through social networks [31,33], but to our knowledge has not been systematically evaluated as a means of providing insight into infectious disease transmission. TE and STE are similar to other model-free methods that measure how information is shared and transferred between possibly coupled dynamic processes, including mutual information [32], Granger causality [34], and convergent cross mapping (CCM) [35]. Permutation entropy, a related measure, has recently been used to quantify the predictability of infectious disease outbreaks [36]. Compared to these alternatives, STE offers the advantage of applying in stochastic settings with nonlinear dynamics and moderate observational noise to reveal asymmetric flows of information.

Here, we use influenza-like outbreak simulations to demonstrate that STE reliably identifies asymmetries in transmission strength between age groups. Then, we use an EMR-based dataset capturing ILI incidence from 884 ZIP (postal) codes and 12 age classes across the USA to rank the relative importance of the various age groups in the transmission of the autumn wave of the 2009 A/H1N1pdm influenza pandemic in that country. We conclude that school-aged children (5–19 year-olds) were disproportionately responsible for transmitting influenza to infants through working-age adults in the autumn of 2009, in broad agreement with other findings. Our work demonstrates that STE could serve as an important tool for the detailed epidemiological analysis of age structure, especially as EMR data become more prevalent.

## 2. Material and methods

### 2.1. Data

The data consist of a convenience sample of CMS-1500 electronic medical claims forms submitted by primary care physicians across the US and maintained by SDI health (now IQVIA). Each claim is associated with a single outpatient visit, and includes one or more ICD-9 codes [37] listed by the physician that describes the patient’s illness. The overall sample captures over 50% of all outpatient visits in the USA in 2009 [17]. The records are binned weekly and aggregated geographically by the first three digits of the ZIP (postal) code of the practice from which they are submitted [38]. Time series of weekly ILI incidence are created by extracting claims with a direct mention of influenza, or fever combined with a respiratory symptom, or febrile viral illness (ICD-9 487-488 OR [780.6 and (462 or 786.2)] OR 079.99), following Viboud *et al.* (2014) [17]. For each ZIP, the number of ILI cases in each week is divided by the total number of patients who visited a physician in that ZIP during that week, yielding an ‘ILI ratio’ time series. There are 884 ILI ratio time series, one for each ZIP in the lower 48 US states, each spanning 52 weeks from the week commencing 4 January 2009 through the week commencing 27 December 2009. The correspondence between the SDI-ILI dataset and reference influenza surveillance data from the US CDC is described in depth by Viboud *et al.* [17].

### 2.2. Symbolic transfer entropy

If *I* and *J* are discrete-state and discrete-time random processes such that *i*_*t*_ and *j*_*t*_ are the states of processes *I* and *J* at time *t*, then the TE from process *J* to process *I* is defined as

2.1 $$T_{J\to I}=\sum_{\Omega_{I},\Omega_{J}}p(i_{t+1},i_{t}^{\left(k\right)},j_{t}^{\left(l\right)})\log(\frac{p(i_{t+1}|i_{t}^{\left(k\right)},j_{t}^{\left(l\right)})}{p(i_{t+1}|i_{t}^{\left(k\right)})}),$$

where $i_{t}^{\left(k\right)}$ is shorthand notation for the *k*-step history of process *i*, (*i*_*t*_, …, *i*_*t*−*k*+1_), and similarly $j_{t}^{\left(l\right)}=(j_{t},\ldots ,j_{t-l+1})$, such that $p(i_{t+1},i_{t}^{\left(k\right)},j_{t}^{\left(l\right)})$ is the joint probability of observing *i*_*t*+1_, $i_{t}^{\left(k\right)}$, and $j_{t}^{\left(l\right)}$; $p(i_{t+1}|i_{t}^{\left(k\right)},j_{t}^{\left(l\right)})$ is the probability of observing *i*_*t*+1_ conditioned on $i_{t}^{\left(k\right)}$ and $j_{t}^{\left(l\right)}$; and $p(i_{t+1}|i_{t}^{\left(k\right)})$ is the probability of observing *i*_*t*+1_ conditioned only on $i_{t}^{\left(k\right)}$. The logarithm has base 2, so that the TE is measured in bits. The sum is over all possible combinations of states $\left(i_{t+1},i_{t}^{\left(k\right)},j_{t}^{\left(l\right)}\right)$, where *i*_*t*+1_, $i_{t}^{\left(k\right)}\in \Omega_{I}$ and $j_{t}^{\left(l\right)}\in \Omega_{J}$, and *Ω*_*I*_ and *Ω*_*j*_ are the state spaces for processes *I* and *J*. Equation (2.1) is a Kullback–Leibler divergence that measures how much process *I* deviates from the generalized Markov property $p(i_{t+1}|i_{t},\ldots ,i_{1})=p(i_{t+1}|i_{t}^{\left(k\right)})$, given the last *l* states of process *J*. In practice, the histories are often fixed at length 1 (*k* = *l* = 1) and the probabilities are estimated from simple counts of the observed data [32].

The TE is limited in that it is only defined for stochastic processes with a discrete state space. Staniek & Lehnertz [31] introduce STE as a way to calculate information transfer between time series processes that have continuous- or near-continuous state spaces. Motivated by the insight that the relative amplitudes of subsequent observations from these sorts of processes may provide enough information to reveal interactions between them, they propose symbolizing the time series based on ordered *m*-tuples of observations (electronic supplementary material, figure S1). This reduces the (near-)continuous state space of the original stochastic process to a discrete set of *m*! symbols. In practice, *m* is often chosen to be 2 or 3, giving a state space of two or six symbols, respectively. For *m* = 3, we also tested the effect of collapsing the two concave-up and the two concave-down symbols into a single symbol each, resulting in a smaller state space (four versus six symbols) while capturing a similar level of qualitative detail. Details on the symbolization of time series and the empirical calculation of the STE are provided in the electronic supplementary material.

### 2.3. SIR epidemic simulation model

For simulations with just two age classes, we use a stochastic SIR model implemented using the Gillespie algorithm [39]. For all simulations, the basic reproduction number *R*_0_ is set at 1.5, consistent with estimates of the basic reproduction number of 2009 A/H1N1 pandemic influenza [40,41]. We consider a population size of *N* = 1000 split evenly between classes 1 and 2, so that *N*_1_ = *N*_2_ = 500 (age groups with different population sizes are also considered in the electronic supplementary material). The expected time to recovery 1/*γ* is assumed constant for all age groups and is set at 7 days, which is consistent with estimates of the infectious period for 2009 pandemic influenza [41]. We consider a range of between-group transmission strengths. Electronic supplementary material, table S1 gives the rates at which individuals of each class stochastically progress from susceptible to infected to recovered. Infections are binned into week-long intervals, and Poisson noise is added to simulate non-influenza ILI. Electronic supplementary material, figure S7 depicts five incidence time series produced using the model. Full details on the model and simulation procedure are given in the electronic supplementary material.

### 2.4. Poisson epidemic simulation model

**Figure 1.**
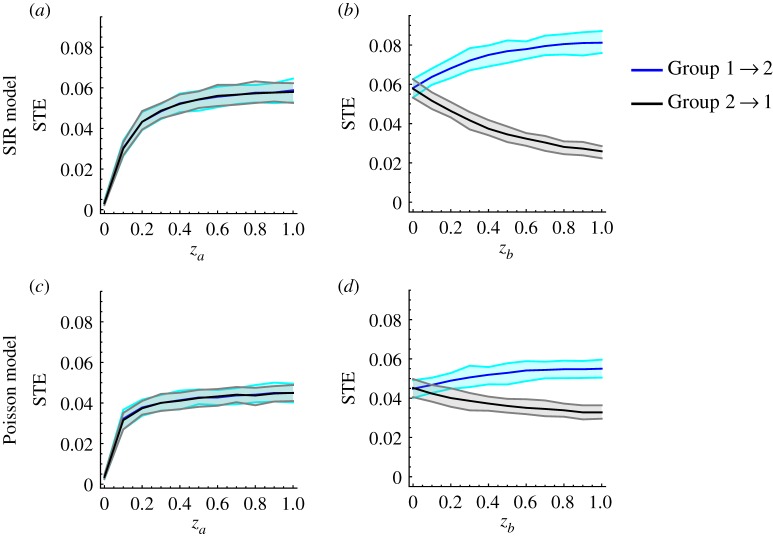
Mean (95% CI) Group 1 → 2 (blue) and Group 2 → 1 (black) STE values as the coupling between the two groups ranges from none to fully symmetric (*a*,*c*), and from fully symmetric to strongly driven by Group 1 (*b*,*d*). The curves are produced by simulating 100 ensembles of 800 epidemics each from the stochastic SIR model (*a*,*b*) or the Poisson model (*c*,*d*) for each value of *z*_*a*_ and *z*_*b*_ between 0 and 1 in steps of 0.1, and then calculating the between-group STE for each ensemble. The relative reproduction matrices that capture these two coupling scenarios are given in equations (3.1) and (3.2).

For more than two age classes, the full stochastic SIR model becomes too computationally demanding for repeated simulations to be practical. So, we also define an outbreak simulation model based on a self-exciting Poisson process, similar to [42]. We choose the time units *t* to match the mean generation interval of the infection, which we set at 3.5 days [43]. To generate epidemics, we use a stepwise-constant effective reproduction number *R*_*t*_, such that *R*_*t*_ = 1.5 for the first four weeks (eight generations) of the outbreak and *R*_*t*_ = 0.8 thereafter. Infections are binned from the half-week generations into week-long intervals, and additional Poisson noise is added to each bin to simulate non-influenza ILI. For simulations with two age classes, the Poisson model yields epidemics of similar length and magnitude as the two-age-class SIR model (compare electronic supplementary material, figures S7 and S8), and yields comparable STE inferences (figure 1), which suggests that the Poisson model is an acceptable approximation to the stochastic SIR model. Full details on the implementation of the Poisson model are given in the electronic supplementary material.

### 2.5. Reporting rates

Only a fraction of influenza cases are represented in the SDI-ILI dataset, since many people do not seek medical care for their symptoms. The tendency to seek medical care given infection with an ILI can vary by age group [44]. To factor this into the outbreak simulations, we introduce a reporting rate vector ***c*** in which element *c*_*i*_ gives the expected proportion of individuals in age class *i* who seek medical care when infected with an ILI. It is then possible to simulate a ‘reported’ disease incidence time series

2.2 $$Y_{i,t}^{\mathrm{obs}}∼\text{binomial}\;(Y_{i,t},c_{i}),$$

where *Y*_*i*,*t*_ is the simulated number of infected individuals in age class *i* at time *t* (under either model) and $Y_{i,t}^{\mathrm{obs}}$ is the simulated reported number of infections in age class *i* at time *t*.

## 3. Results

### 3.1. Symbolic transfer entropy reveals transmission asymmetries between two coupled age groups

We first calculate the STE between two age groups as the within- and between-group reproduction ratios vary. We consider between-group transmission that ranges from (a) fully decoupled to fully symmetric, and (b) fully symmetric to strongly driven by Group 1. The between-group infectiousness is specified using a ‘relative reproduction matrix’ **r**, which is a scaled version of the NGM [27], such that NGM = (*R*_0_/*ρ*)***r***, where *ρ* is the maximum eigenvalue of ***r*** and *R*_0_ is the basic reproduction number. The elements of ***r*** define the relative infectiousness of various population groups, such that **r**_*i*,*j*_/**r**_*k*,*j*_ gives the proportional difference in group *j*’s infectiousness for group *i* versus group *j*. For example, if **r**_*i*,*j*_/**r**_*k*,*j*_ = 2, then a member of group *j* is expected to infect twice as many members of group *i* than of group *k*. The population sizes of the various groups are assumed to be equal, though deviations from this assumption are considered in the electronic supplementary material. Scenario (a) is encapsulated by the relative reproduction matrix

3.1 $$r_{a}=\left[\begin{array}{cc} 1 & z_{a}\\ z_{a} & 1 \end{array}\right],$$

where *z*_*a*_ ∈ [0, 1]. Scenario (b) is encapsulated by the relative reproduction matrix

3.2 $$r_{b}=\left[\begin{array}{cc} 1+3z_{b} & 1\\ 1+z_{b}\; & 1 \end{array}\right],$$

where *z*_*b*_ ∈ [0, 1].

Figure 1 depicts the change in STE under these two transmission scenarios, calculated from epidemics simulated using the stochastic SIR model (figure 1*a*–*b*) and the Poisson model (figure 1*c*–*d*). Each pane in figure 1 is produced using 100 ensembles of 800 simulated epidemics for each value of *z*_*a*_ and *z*_*b*_ between 0 and 1 in steps of size 0.1. For each ensemble, the 800 simulated incidence time series are symbolized using symbols of length *m* = 3, and then the between-group transfer entropies are estimated using the relative symbol frequencies (see electronic supplementary material, figure S3), producing 100 STE estimates for each value of *z*_*a*_ and *z*_*b*_. The solid blue (black) lines in figure 1 depict the mean Group 1 → 2 (Group 2 → 1) STE for each value of *z*_*a*_ and *z*_*b*_ across the 100 ensembles. The shaded blue (black) bands depict the range of the middle 95 Group 1 → 2 (Group 2 → 1) STE estimates for each value of *z*_*a*_ and *z*_*b*_ across the 100 ensembles, analogous to a 95% confidence interval. Under both the stochastic SIR and the Poisson models, the between-group STE increases steadily as the transmission coupling ranges from none to symmetric (figure 1*a*,*c*). Once Group 1 begins to dominate transmission, the Group 1 → 2 STE increases and the Group 2 → 1 STE decreases (figure 1*b*,*d*), accurately capturing the transmission relationship between the age groups.

When Group 1 drives transmission, the Poisson model yields a smaller difference in the STE between the two age groups than the stochastic SIR model does (figure 1*b*,*d*). Visual inspection suggests that the simulated time series produced using the stochastic SIR model tend to feature more stochastic fluctuations than the time series produced using the Poisson model (electronic supplementary material, figures S7 and S8). Since STE is effectively a measure of how these stochastic fluctuations transmit from one age group to another, this may explain why the differences in STE calculated using the Poisson model are relatively less pronounced. Overall, the qualitative similarity between the STE estimates from the two transmission models suggests that the Poisson model is an acceptable approximation to the stochastic SIR model, and that simulations from the Poisson model tend to produce more conservative estimates of the difference in STE between age groups than the stochastic SIR model.

### 3.2. Symbolic transfer entropy reveals transmission asymmetries despite incomplete reporting

**Figure 2.**
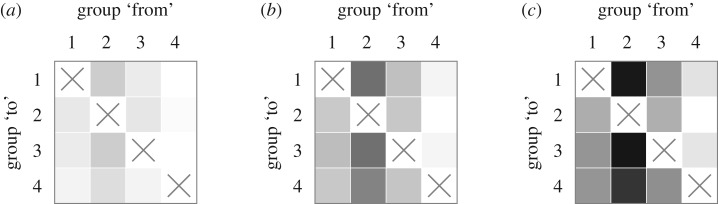
Mean pairwise STE values for epidemics strongly driven by Group 2 with varying reporting rates: (*a*) *c* = 0.1, (*b*) *c* = 0.5 and (*c*) *c* = 1 (see equation (2.2)). A box in row *i* and column *j* corresponds to the STE from group *j* to group *i*, where darker shades corresponds to higher STE. The reporting rate in each subfigure is constant across groups. The STE values are produced by simulating 100 ensembles of 800 epidemics each from the Poisson model for each value of *c* and then calculating the between-group STE for each ensemble. The relative reproduction matrix that specifies within- and between-group transmission rates is given by equation (3.3).

Next, we evaluate how incomplete reporting influences the detection of asymmetries in transmission strength. Figure 2 depicts the mean estimated STE across 100 ensembles of 800 epidemics each for reporting rates *c*_*i*_ = 0.1, *c*_*i*_ = 0.5 and *c*_*i*_ = 1 with equal reporting rates across all age groups (see also electronic supplementary material, figure S9). The epidemic simulations are produced using the Poisson model with relative reproduction matrix

3.3 $$r=\left[\begin{array}{cccc} 1 & 2 & 1 & 1\\ 1 & 4 & 1 & 1\\ 1 & 2 & 1 & 1\\ 1 & 1 & 1 & 1 \end{array}\right],$$

which could represent ‘children’ (Group 2) having strong within-group transmission (*r*_2,2_ = 4) and intermediate transmission to ‘infants’ (Group 1) and ‘adults’ (Group 3) (*r*_1,2_ = *r*_3,2_ = 2). Even for reporting rates as low as 0.1, the STE values from Group 2 are higher than those from any other group. As the reporting rates increase, the differences become more pronounced, accurately capturing the transmission dominance of Group 2 over the other groups. According to Biggerstaff *et al.* [44], true reporting rates for ILI in the US during the 2009 pandemic were between 0.4 and 0.6 (scenario (B) in figure 2), for which the transmission dominance of Group 2 is clear.

### 3.3. Symbolic transfer entropy reveals transmission asymmetries between 12 coupled age groups

To test the ability of STE to identify transmission asymmetries from data on the scale of the SDI-ILI dataset, we use the Poisson model to simulate 100 ensembles of 800 epidemics each with 12 age groups. We consider the scenarios (a) with the 12 × 12 relative reproduction matrix electronic supplementary material, Eq. S49, representing high transmission from Groups 3–5 to Groups 3–5 (*r*_*i*,*j*_ = 4 for *i*, *j* ∈ {3, 4, 5}), intermediate transmission from groups 3–5 to groups 1–2 and 6–9 (*r*_*i*,*j*_ = 2 for *i* ∈ {1, 2, 6, 7, 8, 9} and *j* ∈ {3, 4, 5}), baseline transmission (*r*_*i*,*j*_ = 1) between all other groups, and uniform 50% reporting rate across all groups, and (b) with uniform transmission strength across all age groups (i.e. a 12 × 12 relative reproduction matrix with ‘1’ for all entries), 60% reporting rate for groups 1–5, and 40% reporting rate for groups 6–12, following the estimates of Biggerstaff *et al.* [44] for the ILI reporting rates in the USA during the 2009 influenza pandemic for children and adults, respectively.

**Figure 3.**
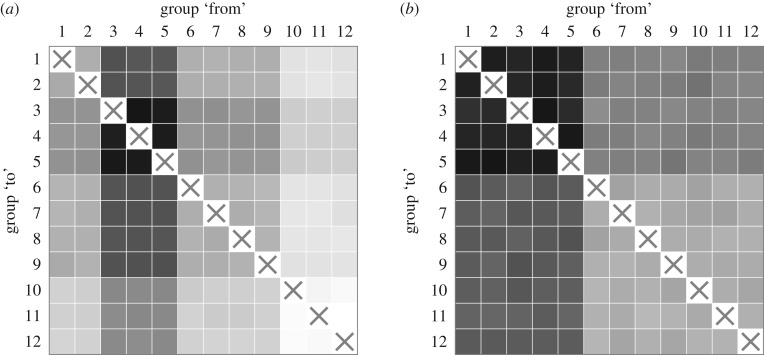
Mean pairwise STE values between 12 groups for epidemics strongly driven by Groups 3, 4 and 5 and uniform 50% reporting rate across all age groups (*a*), and for epidemics driven equally by all age groups, 60% reporting for Groups 1–5, and 40% reporting for Groups 6–12 (*b*). A box in row *i* and column *j* corresponds to the STE from group *j* to group *i*, where darker shades corresponds to higher STE. To generate the STE values, 100 ensembles of 800 epidemics were simulated from the Poisson model using relative rate matrix electronic supplementary material, Eq. S49 for (*a*) or a relative rate matrix with all entries equal to 1 for (*b*). Each ensemble generates 144 pairwise STE values, so that each box represents the mean value across the 100 ensembles. The raw values are listed in electronic supplementary material, Eqs S50 and S51.

Figure 3 depicts the mean pairwise STE estimates between the 12 age groups under both scenarios. The square in row *i* and column *j* represents the STE from Group *j* to Group *i*. Darker squares correspond to higher STE. For the asymmetric transmission/uniform reporting rate scenario (scenario (a), figure 3*a*), the STE clearly captures the transmission dominance of Groups 3, 4 and 5. The pairwise STE does not simply reproduce the structure of the relative reproduction matrix, as evidenced by the variability in mean pairwise STE for age groups other than Groups 3–5. This is because the STE captures a ‘knock-on’ effect for which information transferred from a strongly driving age group can propagate through other age groups. For the uniform transmission/variable reporting rate scenario (scenario (b), figure 3*b*), it is evident that elevated reporting rates can also lead to elevated STE, both to and from the groups with elevated reporting rate (Groups 1–5). Overall, the variability in STE due to differences in reporting rate appears to be smaller than the variability in STE due to differences in transmission strength. Further discussion on the effect of reporting rates on STE may be found in the electronic supplementary material.

### 3.4. School-aged children contributed disproportionately to transmission during the autumn 2009 A/H1N1pdm influenza outbreak in the US

**Figure 4.**
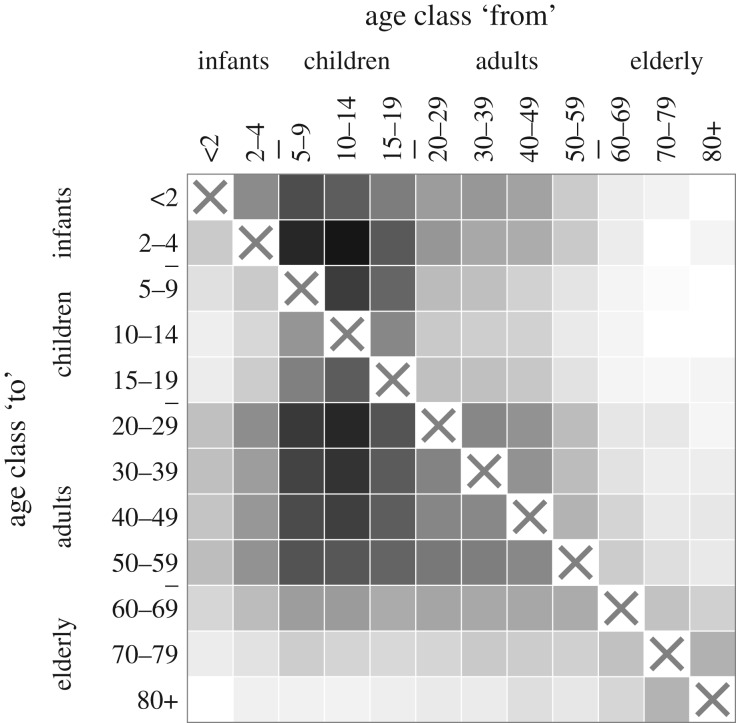
Mean pairwise STE values between the 12 groups represented in the SDI-ILI dataset during the autumn 2009 A/H1N1pdm pandemic influenza outbreak. A box in row *i* and column *j* corresponds to the STE from group *j* to group *i*, where darker shades corresponds to higher STE. The raw values are listed in electronic supplementary material, Eq. S52.

To estimate the pairwise STE between the 12 age groups represented in the SDI-ILI dataset during the 2009 A/H1N1pdm influenza pandemic, we extract data from the 25 weeks between 12 July 2009 and 27 December 2009 and symbolise the ILI time series for each age group in each ZIP using a symbol length of *m* = 3. The pairwise STE values between all age groups are depicted in figure 4. The STE is highest in the columns representing 5–19 year-olds. This provides evidence that there was systematically elevated transmission from school-aged children to infants through adults. The adult-adult STE is also moderately elevated, suggesting that adults may have played a relatively important role in transmitting the outbreak among themselves, though this could also be explained by elevated transmission from children alone. Compare, for example, to the left-hand plot in figure 3: in that simulation, only transmission from children is elevated, but it causes a moderate elevation in the STE from adults and infants to the other age groups due to the knock-on effect.

As a control, we also calculated the pairwise STE between all age groups during 25 post-pandemic weeks, from 10 January 2010 through 27 June 2010. For these months, there is no apparent age structure in transmission (see electronic supplementary material). We also calculated the pairwise STE between age groups for six previous influenza seasons (see electronic supplementary material). For the 2009 pandemic, there is a higher maximum pairwise STE and greater variation in the pairwise STEs than for any previous season. This could reflect differences in baseline ILI, which was likely lower during the autumn 2009 pandemic wave than during the seasonal outbreaks, due to the pandemic’s earlier timing. A lower baseline ILI might have made pairwise differences in STE easier to detect in 2009. However, the relatively higher and more heterogeneous STE values in 2009 are also consistent with the hypothesis that school-aged children played a disproportionately large role in the spread of the 2009 pandemic, as has been described elsewhere [4].

It is unlikely that differences reporting rates alone can account for the elevated STE from 5 to 19 year-olds to the other age groups. The mean pairwise STE values computed from simulations with uniform transmission rates and unequal reporting rates in §3.3 range from 0.0057 to 0.0084 (see electronic supplementary material, Eq. S51), while the pairwise STE values computed from the SDI-ILI data range from 0.0056 to 0.084 (see electronic supplementary material, Eq. S52), an order of magnitude larger. The mean pairwise STE values computed from simulations with asymmetric transmission and uniform reporting rates §3.3 range from 0.0047 to 0.014 (see electronic supplementary material, Eq. S50), closer to the range observed from the SDI-ILI data but still somewhat smaller. This points towards a possible combined effect of strong transmission driving from children plus elevated reporting in children. In addition, re-calculating the pairwise STE using probabilistic reconstructions of the pre-reporting SDI-ILI incidence time series (see electronic supplementary material) indicate that the observed transmission dominance of 5–19 year-olds persists even after adjusting for potential differences in reporting rate between children and adults. Furthermore, Biggerstaff *et al.* [44] report that 0–4 year-olds had the highest reporting rates for ILI in the USA in 2009, yet the STE from 0 to 4 year-olds is relatively low compared to the other age groups. If reporting rates alone could explain the observed differences in STE, the STE from infants should be at least as high as the STE from school-aged children.

It is also unlikely that the unequal partitions of the age groups can explain the observed patterns in the pairwise STE. The age groups under 20 years are partitioned such that they span fewer years, and thus contain fewer individuals, than the age groups above 20 years. Direct calculations and simulations (see electronic supplementary material) indicate that, all else being equal, the out-going STE for a given group tends to increase as the group’s population size increases relative to the sizes of the other groups. If differences in the groups’ population sizes were driving the observed pairwise STE values, we would expect the age groups over 20 years to appear to dominate transmission—which is the opposite of what we observe here.

## 4. Discussion

Here, we propose STE as a means of ranking which age groups contribute most to the transmission of infectious disease outbreaks. STE is chosen over other extensions of TE due to its robustness to point-wise noise and overall amplitude shifts in time series, which especially affect the ILI data stream due to non-influenza respiratory illness and incomplete reporting. Simulation studies indicate that STE can correctly rank transmission asymmetries between age groups. While such a ranking does not provide definitive guidance for targeted interventions, it can provide a useful starting point when other epidemiological information is lacking, as is often the case in emerging outbreaks. STE is positively associated with reporting rates, which can partially confound estimates of asymmetric transmission. STE estimates from ILI time-series data from July–December 2009 in the USA suggest that the transmission of the autumn wave of the A/H1N1pdm pandemic influenza outbreak was likely dominated by 5–19 year-olds. It is unlikely that this result can be explained by differences in reporting rates alone.

The identification of elevated transmission from school-aged children during the 2009 influenza pandemic agrees with most other studies on age-specific transmission of both seasonal and pandemic influenza [1,4,7,8]. Elevated transmission from school-aged children is likely due in part to the relatively high number of daily interpersonal contacts made by members of these age groups. Mossong *et al.* [1] for example estimate that 10–19 year-olds have more contacts per day than any other age group, and conclude from a modelling study based on empirical contact data that 5–19 year-olds are likely to both suffer the highest burden of disease and to drive the early-stage transmission of an outbreak transmitted by droplets through close contacts, like influenza. This underscores the importance of monitoring children during pandemic influenza outbreaks. We do not recommend using STE alone to set vaccination priorities. STE is shown here to reveal the population structure of transmission, but as a correlation-based measure [45], it does not provide conclusive evidence that vaccinating high-transmission groups would optimally reduce overall transmission. If high-specificity (e.g. laboratory-confirmed) data are available for a subset of the population, we recommend using STE in tandem with existing risk-based methods [4,29,30] to identify optimal intervention strategies. For example, one might use STE to identify epidemiologically relevant partitions of the population (for the 2009 A/H1N1pdm influenza outbreak in the US, these might be <5, 5–19, 20–59, and 60+ years). Then, using these demographic partitions, one could calculate the relative risk of infection between these coarser groups from the high-specificity data, thereby leveraging the data to its fullest potential.

In this study, we have only considered a single demographic variable—age—as the basis for examining asymmetric transmission strengths. Age is known to be a key predictor of influenza transmission [1,7,8]. For other infectious diseases, different demographic variables will be necessary. In the context of sexually transmitted infections, for example, one might consider transmission asymmetries between individuals of different racial/ethnic [46] or sexual behaviour [47] groups. Our study supports the hypothesis that among all age groups, but not necessarily among all possible demographic groups, school-aged children were the strongest transmitters of influenza during the autumn 2009 A/H1N1pdm outbreak in the US.

TE is closely linked to mutual information [32] and Granger causality [34]. Unlike TE, mutual information is symmetric; that is, it measures the probabilistic dependence between two processes, but cannot determine the direction of information transfer between them, if there is any [32]. Measuring the delayed mutual information between two processes is one way to introduce asymmetry. This takes a step towards inferring whether one process influences another, by measuring shared information between the present state of one process and the past states of another [32]. While the lagged mutual information describes how one process history predicts the static probabilities of another, the TE measures how one process history predicts the transition probabilities of another. Because of this, the TE is less likely to be confounded by a shared input signal, and is a better measure of stochastic ‘driving’ [32]. Section 2 of Kaiser & Schreiber [48] provides a detailed description of the differences between TE and mutual information. Granger causality, on the other hand, is a special case of TE that arises when the stochastic processes are jointly Gaussian distributed [49]. The TE is thus better suited than Granger causality for making inferences on more general, possibly nonlinear, processes, though this comes at the expense of requiring more data and having no clear way to test statistical significance [49].

CCM [35] was developed to solve a similar problem as TE, but is based on somewhat different underlying theory. CCM was developed to detect so-called causal relationships in partially stochastic systems with underlying deterministic structure. CCM relies on Takens’ theorem [50] to reconstruct candidate manifolds of the underlying dynamical system using lagged observations from two-time series. ‘Causality’ is inferred if nearby points on one reconstructed manifold consistently map to nearby points on the other reconstructed manifold. CCM has been used to provide evidence that temperature and absolute humidity fluctuations drive the timing of global seasonal influenza outbreaks [51], though some controversy surrounds these findings [52,53]. Nevertheless, it would be interesting to see whether CCM can reveal asymmetric epidemiological interactions between age groups, and to compare its findings with those identified using TE. Lungarella *et al.* [54] provide more detail on the relationships between various methods that infer asymmetric relationships from time-series data. As an aside, we prefer to avoid the term ‘causality’ with respect to these methods, despite its frequent use in the literature. Determination of so-called counterfactual causality, as distinguished from Granger-type causality, requires intervention [55], which is normally not possible in retrospective epidemiological studies. Regardless of the vocabulary used, the above-listed techniques have successfully revealed fundamental structures in real-world coupled dynamic processes [31,33,35,56–58].

Despite the apparent well-suitedness of STE for making inferences from ILI data, its epidemiological relevance currently remains limited. The calculation of STE requires no prior epidemiological information whatsoever, which makes its success somewhat surprising. The NGM [27] is the key object for characterizing age-structured, or more generally population-structured, disease transmission dynamics, and yet there is no obvious direct link between STE estimates and the NGM. It is possible that further simulation studies could help identify such a link; even though the STE values seem to bear little mechanistic meaning apart from the relative ordering of age groups that they yield, it is possible that regressing the inferred STE values on an underlying known NGM could connect the pairwise STE matrix with the NGM under certain conditions. However, it appears unlikely that a simple link exists, especially since STE can say nothing about transmission within a single age group, which is necessary for filling in the diagonal entries of the NGM. STE and related methods such as CCM that do not explicitly incorporate mechanistic descriptions of the underlying physical system are unlikely to be able to reveal more than an approximate hierarchy of transmission strengths. Nevertheless, such a hierarchy can contain valuable information, especially if developing and fitting a mechanistic model is too demanding to be practicable. Certain extensions to STE could also enhance its relevance for epidemiological inference. Local TE [59] and state-dependent TE [60], like the contextual STE (see electronic supplementary material), are intended to make the TE more flexible and general, by considering how information transfer may change under varying conditions or ‘meta-states’. Conditional TE [45,61] takes fuller account of the possible drivers of a given signal which could help to reveal polyadic and synergistic relationships between demographic groups. These extensions may yield better insight into epidemic processes, which are inherently nonlinear and context-dependent, than the more traditional measurements of TE can provide.

Perhaps the most important challenge confronting the TE and related measurements is deciding how to measure statistical power and significance. STE calculations rely on a middle level of stochasticity in the underlying stochastic processes; for a deterministic system, the STE will always be exactly zero, while for a stochastic system with too much within-sequence noise, the small-scale variation in amplitudes will likely mask important patterns from which the transfer of information might be inferred. The acceptable range of stochasticity has not been clearly defined. Similarly, it is unclear how best to measure when a difference in STE should be called statistically significant. Though this is recognized as an open and difficult problem [49,52], it may be possible to make some progress by assuming that the underlying process follows certain epidemiological, or otherwise well-specified, dynamics.

## Supplementary Material

Supplemental Materials and Methods

## Data accessibility

The data used in this article and the corresponding access requirements are described in ‘Spatial Transmission of 2009 Pandemic Influenza in the US’, by J. R. Gog *et al.* (2014), *PLOS Computational Biology* [62].
