# Supplementary material for: Symbolic transfer entropy reveals the age structure of pandemic influenza transmission from high-volume influenza-like illness data
Source: J R Soc Interface. 2020 Mar 18;17(164):20190628. doi: 10.1098/rsif.2019.0628 (PMC7115222; doi:10.1098/rsif.2019.0628)
Supplement: Supplemental Materials and Methods [file rsif20190628supp1.pdf]

SUPPLEMENTAL INFORMATION FOR:
"USING SYMBOLIC TRANSFER ENTROPY TO INFER WHICH AGE GROUPS DRIVE INFLUENZA TRANSMISSION FROM INFLUENZA-LIKE ILLNESS DATA"

SM KISSLER, C VIBOUD, BT GRENFELL, JR GOG

1. Calculating the STE

The symbolisation strategy that underlies symbolic transfer entropy (STE) [1] captures the qualitative structure of an observed time series by reducing the time series $\{i_t\}$ with $i_t \in \mathbb{R}$ and $t \in 1, 2, 3, \ldots, n$ to a sequence of symbols that represent ordered m-tuples of observations. The modeller chooses the number of consecutive points m that make up each symbol. For $m = 2$, there are only two possible symbols: $i_{t-1} < i_t$ and $i_{t-1} > i_t$. For $m = 3$, there are six possible symbols, which we label A through F:

$$A: \ i_{t-2} < i_{t-1} < i_t \tag{S1}$$
$$B: \ i_{t-2} < i_t < i_{t-1} \tag{S2}$$
$$C: \ i_{t-1} < i_{t-2} < i_t \tag{S3}$$
$$D: \ i_{t-1} < i_t < i_{t-2} \tag{S4}$$
$$E: \ i_t < i_{t-2} < i_{t-1} \tag{S5}$$
$$F: \ i_t < i_{t-1} < i_{t-2} \tag{S6}$$

These symbols are depicted in Fig S1. In general, for a given m, there are $m!$ possible symbols. For a stochastic process I with n ordered observations $(i_1, \ldots i_n)$, a symbol is assigned to observations $(i_m, i_{m+1}, \ldots, i_n)$. Fig S2 depicts how a given time series would be symbolised for $m = 2$ and $m = 3$. Doing so yields a new process $\hat{I}$ with observations $(\hat{i}_m, \hat{i}_{m+1}, \ldots, \hat{i}_n)$. The same may be done for a second process J. The symbolic transfer entropy (STE) is then defined as

$$T^S_{J \to I} = \sum p(\hat{i}_{t+1}, \hat{i}_t, \hat{j}_t) \log\Big(\frac{p(\hat{i}_{t+1}|\hat{i}_t, \hat{j}_t)}{p(\hat{i}_{t+1}|\hat{i}_t)}\Big) \tag{S7}$$

where the sum is over all possible symbols for states $\hat{i}_{t+1}$, $\hat{i}_t$, and $\hat{j}_t$.

In practice, the joint and conditional probabilities in Eq. S7 are estimated using the relative observed frequencies of the symbols. For example, to calculate the joint probability $p(\hat{i}_{t+1} = A, \hat{i}_t = B, \hat{j}_t = C)$ for symbols A, B, and C, one counts the number of times a consecutive B and then A are observed in the I process, with a C in the J process simultaneous with the B in the I process. Dividing this count by the total length of the process, $n - m + 1$, gives an estimate of the joint probability.

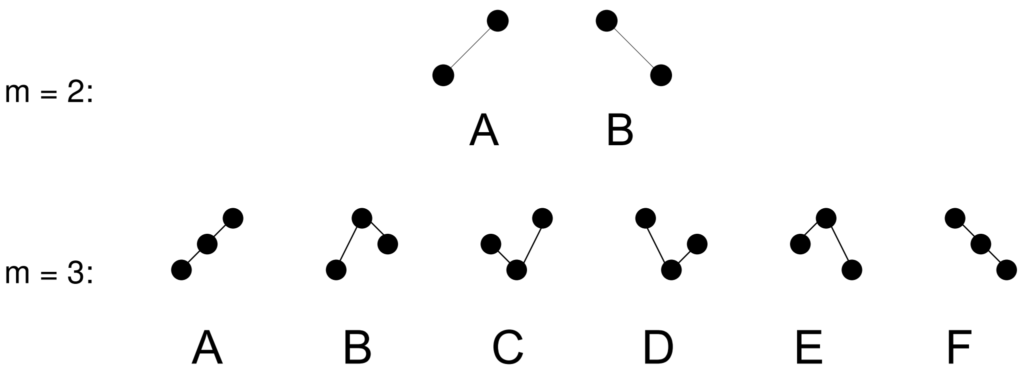

FIGURE S1. List of the two possible symbols for $m = 2$ (top) and the six possible symbols for $m = 3$ (bottom). The six $m = 3$ symbols are specified by Eqs S1-S6. The ordering of the symbols is arbitrary, but will remain consistent throughout this text.

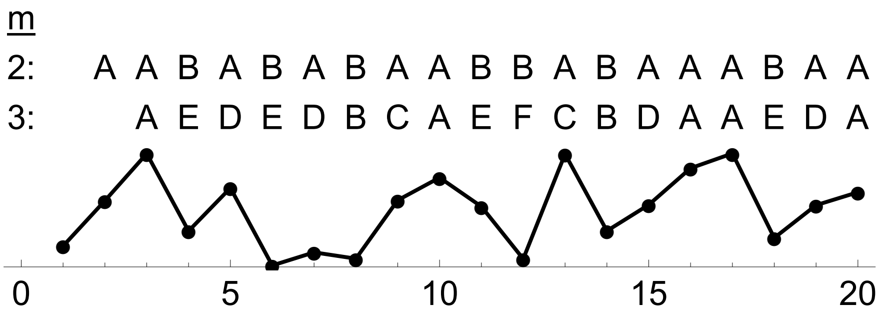

FIGURE S2. An example time series and its symbolisation with $m = 2$ and $m = 3$. For $m = 2$, A represents an increase and B represents a decrease. The symbols corresponding to the letters for $m = 3$ are defined in Eq. S1-S6 and depicted in Fig S1.

If multiple realisations of the process are available, the probabilities may be estimated by the relative frequencies of the symbols across all realisations. The process need not have reached a stationary distribution, as long as enough realisations are available [2]. Fig S3 depicts how this is done. Consider k realisations from two possibly related stochastic processes I and J. Each realisation consists of a time series of length n. So, there are k pairs of length-n time series available for estimating the STE between variables I and J. In Fig S3, $k = 3$ and $n = 17$. To estimate the STE, each of the time series is symbolised using symbols of length m. This yields k pairs of symbol strings, where each string within a pair has length $n_S = n - (m - 1)$. In Fig S3, $m = 2$, so each symbol string has length $n_S = 17 - (2 - 1) = 16$.

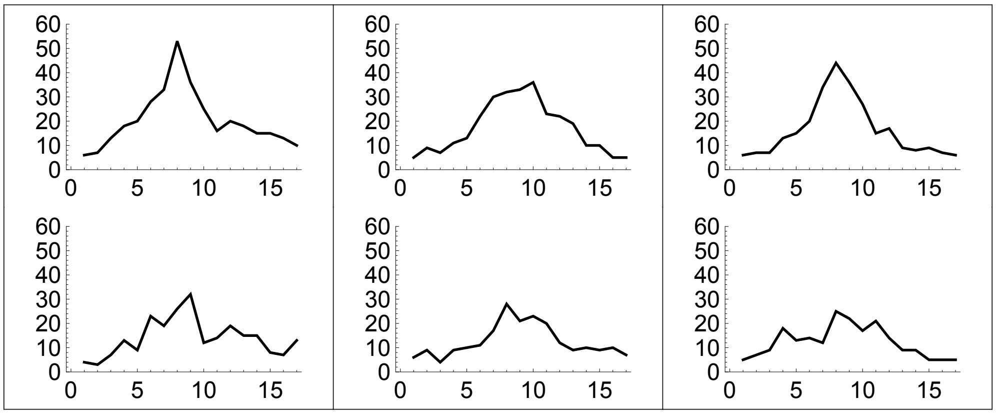

AAAAAAABBBABBBBB ABAAAAAAABBBBBBB ABAAAAABBBABBABB

BAABABAABAABBBBA ABAAAAABABBBABAB AAABABABBABBBBBB

$$P(\hat{i}_{t+1} = B, \hat{i}_t = A, \hat{j}_t = A) = \frac{2+2+2}{15+15+15} = \frac{6}{45}$$

$$P(\hat{i}_{t+1} = B \mid \hat{i}_t = A, \hat{j}_t = A) = \frac{2+2+2}{5+7+4} = \frac{6}{16}$$

$$P(\hat{i}_{t+1} = B \mid \hat{i}_t = A) = \frac{2+2+4}{8+8+8} = \frac{8}{24}$$

$$\vdots$$

FIGURE S3. Summary of how the STE is calculated from multiple realisations of an epidemic process with two age groups. The three columns in the top pane portray $k = 3$ realisations from an epidemic process, which in this case is the Poisson-type process described in the main text. The columns consist of two time series plots each, which depict the simulated case counts for the two age groups (call them I and J) across 17 time steps. The time series are symbolised with a symbol length of $m = 2$, yielding the character strings beneath the first set of arrows. An A represents an increase and a B represents a decrease. The probabilities that make up the STE calculation, Eq. S7, are estimated using the relative frequencies of the symbols in the character strings. A few of these probabilities are calculated in the lower section of this figure.

Next, the probabilities that make up the STE calculation, Eq. S7, are estimated using the relative frequencies of the symbols in the symbol strings. For example, $P(\hat{i}_{t+1} = B, \hat{i}_t = A, \hat{j}_t = A)$ is calculated as the probability that there is a B in the I process that is preceded by an A in the I process and also an A in the J process. It is helpful to interpret this as the number of times the pattern

$$\begin{matrix} A & B \\ A & \end{matrix} \tag{S8}$$

is observed when the symbol strings for a realisation of I and J are aligned one above the other (see the symbol strings in Fig S3). The frequency with which this pattern appears is calculated for each of the k pairs of symbol strings, yielding a set of counts $c_1, \ldots, c_k$. Since there are $n_S - 1$ possible positions for the pattern to appear within each pair of symbol strings, the overall joint probability is estimated as

$$\frac{c_1 + c_2 + \cdots + c_k}{k(n_S - 1)}. \tag{S9}$$

This fraction is interpreted as the number of times a given 'triad' of symbols (*e.g.* Eq. S8) is observed across all realizations, divided by the total number of times the triad could have possibly occurred.

Conditional probabilities are estimated in a similar way. For example, $P(\hat{i}_{t+1} = B | \hat{i}_t = A, \hat{j}_t = A)$ is estimated as the number of times a B is observed in the I process, given that the previous observations in the I and J processes were both A. The counts $c_1, \ldots, c_k$ are the same as before — we are seeking the same triad of symbols — but rather than dividing by the total length of the symbolised time series, we instead divide by the number of times the pattern

$$\begin{matrix} A \\ A \end{matrix}$$

is observed. Denote these counts $\hat{c}_1, \ldots, \hat{c}_k$. Then, the conditional probability is

$$\frac{c_1 + c_2 + \cdots + c_k}{\hat{c}_1 + \hat{c}_2 + \cdots + \hat{c}_k}. \tag{S10}$$

The calculation is easier for the probabilities conditioned on just one term. The quantity $P(\hat{i}_{t+1} = B | \hat{i}_t = A)$, for example, is simply the number of times an AB appears in the symbolised time series for I, divided by the number of times an A appears in the symbolised time series for I.

The above steps are demonstrated with a specific example in Fig S3. The joint and conditional probabilities in Eq. S7 may be estimated using the steps outlined above, adjusting the specific symbols as necessary.

2. The Contextual STE

The STE assumes that that there are Markovian transitions between the symbols of the symbolised time series – that is, the probability of observing a particular symbol, given the symbol that precedes it (and possibly given the preceding value in some other time series), is some constant value, regardless of the exact amplitude of the underlying process. However, for disease outbreaks, the amplitude of the underlying process (i.e. the case count) clearly does matter. For an outbreak with case counts binned into regular time windows, the probability that an increase in cases occurs in a given age group from one time step to the next is related to the number of cases in each age group in the previous time step, the overall reproduction number, and the relative rates of infection between age groups. When the reproduction number is greater than 1, an increase in cases is more likely if there are, for example, 1000 cases in the current week than if there are 10. In the vocabulary of STE, when using symbols of length $m = 2$ (thus encoding simple increases/decreases in the time series), the probability of transitioning from the symbol $i_t < i_{t+1}$ to the (same) symbol $i_{t+1} < i_{t+2}$ is higher when i_t is 1000 than when i_t is 10, if the reproduction number is greater than 1. This violates the Markovian assumption embedded into the STE calculation, which assumes that the joint and conditional probabilities in Eq S7 are consistent throughout the entire process.

To test whether the STE can provide reliable information about age-structured transmission dynamics despite this deviation from the Markov property, we here calculate a version of the STE for a range of epidemiologically reasonable reproduction numbers, between-age-group infection rates, and true numbers of infected individuals. To lay the groundwork, consider an outbreak where the number of cases per unit time in two age groups are described by the discrete random processes $I = \{i_1, \ldots, i_n\}$ and $J = \{j_1, \ldots, j_n\}$. Define the contextual STE as

$$T^S_{J \to I,t} = \sum p(\hat{i}_{t+1}, \hat{i}_t, \hat{j}_t | i_{t-m+1}, j_{t-m+1}) \log\Big(\frac{p(\hat{i}_{t+1}|\hat{i}_t, \hat{j}_t, i_{t-m+1}, j_{t-m+1})}{p(\hat{i}_{t+1}|\hat{i}_t, i_{t-m+1}, j_{t-m+1})}\Big). \tag{S11}$$

where the sum is over all possible values (symbols) of $\hat{i}_{t+1}$, $\hat{i}_t$, and $\hat{j}_t$, and m is the symbol length. Values with hats denote symbols, and values without hats denote actual case counts. The contextual STE is equivalent to the full STE (Eq S7), conditional on the true amplitudes of processes I and J at time $t - m - 1$, or the 'context' of the underlying process. If the contextual STE gives accurate insight into the underlying epidemiological process regardless of the context, then there is reason to believe that the full STE will give similarly accurate insight.

Next, we define an epidemiological model that describes the distribution of cases in age groups I and J at (discrete) time t, given the number of cases in each age group at time $t - 1$:

$$P(i_t|i_{t-1}, j_{t-1}) = f^I(i_t; i_{t-1}, j_{t-1}, \boldsymbol{\lambda}) \tag{S12}$$

$$P(j_t|i_{t-1}, j_{t-1}) = f^J(j_t; i_{t-1}, j_{t-1}, \boldsymbol{\lambda}) \tag{S13}$$

where f^I and f^J are probability mass functions, and $\boldsymbol{\lambda}$ is a set of parameters specifying the rates of infection between age groups I and J. The case counts i_t and j_t are assumed to be nonnegative integers. We first simplify notation by setting

$$f^I(i_t; i_{t-1}, j_{t-1}, \boldsymbol{\lambda}) = f^I_{t-1}(i_t) \quad \text{and} \tag{S14}$$

$$f^J(j_t; i_{t-1}, j_{t-1}, \boldsymbol{\lambda}) = f^J_{t-1}(j_t) \tag{S15}$$

and similarly defining the corresponding CDFs

$$F^I_t(x) = \sum_{k=0}^{x} f^I_t(k) \tag{S16}$$

$$F^J_t(x) = \sum_{k=0}^{x} f^J_t(k) \tag{S17}$$

where x is a nonnegative integer.

A common choice is to model the disease case counts as Poisson random variables, each with a rate that is a linear combination of the case counts in the previous time step in each age group:

$$P(i_t|i_{t-1}, j_{t-1}) \sim Poisson(\lambda_{11} i_{t-1} + \lambda_{12} j_{t-1}) \tag{S18}$$

$$P(j_t|i_{t-1}, j_{t-1}) \sim Poisson(\lambda_{21} i_{t-1} + \lambda_{22} j_{t-1}) \tag{S19}$$

giving

$$f^I_{t-1}(i_t) = \text{Exp}[-(\lambda_{11} i_{t-1} + \lambda_{12} j_{t-1})] \frac{(\lambda_{11} i_{t-1} + \lambda_{12} j_{t-1})^{i_t}}{i_t!} \quad \text{and} \tag{S20}$$

$$f^J_{t-1}(j_t) = \text{Exp}[-(\lambda_{21} i_{t-1} + \lambda_{22} j_{t-1})] \frac{(\lambda_{21} i_{t-1} + \lambda_{22} j_{t-1})^{j_t}}{j_t!}. \tag{S21}$$

If the length of the time steps matches the disease's generation interval, the next-generation matrix is simply

$$NGM = \begin{bmatrix} \lambda_{11} & \lambda_{12} \\ \lambda_{21} & \lambda_{22} \end{bmatrix}, \tag{S22}$$

and the reproduction number is the dominant eigenvalue of this matrix.

We now seek to express the contextual STE, Eq S11, in terms of the epidemiological process, defined by f^I and f^J. For now, consider a symbol length of $m = 2$, and hence $2! = 2$ symbols. The contextual STE sum has $2 \times 2 \times 2 = 8$ terms, one for each set of possible symbols $\{\hat{i}_{t+1}, \hat{i}_t, \hat{j}_t\}$. The derivation will be done for a single term of that sum. The derivations for the other terms follow nearly identical steps.

Consider the term of Eq S11 corresponding to two consecutive decreases in process I with a concurrent decrease in process J:

$$\text{(S23)} \quad P(i_{t+1} < i_t, i_t < i_{t-1}, j_t < j_{t-1} | i_{t-1}, j_{t-1}) \log\Big(\frac{P(i_{t+1} < i_t | i_t < i_{t-1}, j_t < j_{t-1}, i_{t-1}, j_{t-1})}{P(i_{t+1} < i_t | i_t < i_{t-1}, i_{t-1}, j_{t-1})}\Big)$$

where the inequalities now take the place of the symbols (the terms with hats) from Eq S11. We seek to express these probabilities in terms of f^I and f^J. To avoid cluttering notation, assume that i_{t-1} and j_{t-1} are given throughout. First, consider the joint probability (the first term, before the logarithm in Eq S23):

$$\text{(S24)} \quad P(i_{t+1} < i_t, i_t < i_{t-1}, j_t < j_{t-1}) =$$

$$\text{(S25)} \quad P(i_t < i_{t-1}, j_t < j_{t-1}) P(i_{t+1} < i_t | i_t < i_{t-1}, j_t < j_{t-1}) =$$

$$\text{(S26)} \quad P(i_t < i_{t-1}) P(j_t < j_{t-1}) P(i_{t+1} < i_t | i_t < i_{t-1}, j_t < j_{t-1}) =$$

$$\text{(S27)} \quad P(i_t < i_{t-1}) P(j_t < j_{t-1}) \sum_{j_t=0}^{j_{t-1}} \sum_{i_t=0}^{i_{t-1}} P(i_{t+1} < i_t | j_t, i_t) P(i_t | i_t < i_{t-1}) P(j_t | j_t < j_{t-1}) =$$

$$\text{(S28)} \quad = F^I_{t-1}(i_{t-1}) F^J_{t-1}(j_{t-1}) \sum_{j_t=0}^{j_{t-1}} \sum_{i_t=0}^{i_{t-1}} F^I_t(i_t) \frac{f^I_{t-1}(i_t)}{F^I_{t-1}(i_{t-1})} \frac{f^J_{t-1}(j_t)}{F^J_{t-1}(j_{t-1})} =$$

$$\text{(S29)} \quad = \sum_{j_t=0}^{j_{t-1}} \sum_{i_t=0}^{i_{t-1}} F^I_t(i_t) f^I_{t-1}(i_t) f^J_{t-1}(j_t)$$

where (S25) follows from a definition of conditional probability, (S26) follows from the independence of i_t and j_t given i_{t-1} and j_{t-1}, (S27) follows from the law of total probability, (S28) follows from substituting terms, and (S29) follows from cancellation of the two CDFs in I and J.

The probabilities inside the logarithm in Eq S23 may be similarly expressed in terms of the epidemiological process, giving

$$\text{(S30)} \quad \sum_{j_t=0}^{j_{t-1}} \sum_{i_t=0}^{i_{t-1}} F^I_t(i_t) f^I_{t-1}(i_t) f^J_{t-1}(j_t) \log\Big(\frac{\sum_{j_t=0}^{j_{t-1}} \sum_{i_t=0}^{i_{t-1}} F^I_t(i_t) f^I_{t-1}(i_t) f^J_{t-1}(j_t)}{F^J_{t-1}(j_{t-1}) \sum_{j_t=0}^{\infty} \sum_{i_t=0}^{i_{t-1}} F^I_t(i_t) f^I_{t-1}(i_t) f^J_{t-1}(j_t)}\Big)$$

as an alternative expression for the term (S23). The remaining seven terms in the contextual STE sum, Eq S11, may also be expressed in terms of the epidemiological process using similar derivations. It is now possible to explore how different epidemiological scenarios affect the contextual STE.

2.1. **Contextual STE under various epidemiological scenarios.** This section considers how the contextual STE varies with reproduction number R and underlying case counts i_{t-1} and j_{t-1}, given some relative within- and between-group rates of transmission.

First, note that the contextual STE from group J to group I is zero when the number of cases in group I does not depend on the previous number of cases in group J; that is, when $P(i_t | i_{t-1}, j_{t-1}) = P(i_t | i_{t-1})$. This property should be expected, since there is no transfer of infection, and thus should

be no transfer of information, from group J to group I. To verify, consider the numerator in the logarithm in Eq S30. Since F_t^I and f_{t-1}^I do not depend on j_t, the sum over j_t can be brought in front of the final term, yielding

$$\sum_{i_t=0}^{i_{t-1}} F_t^I(i_t) f_{t-1}^I(i_t) \sum_{j_t=0}^{j_{t-1}} f_{t-1}^J(j_t) \tag{S31}$$

$$= \Big(\sum_{i_t=0}^{i_{t-1}} F_t^I(i_t) f_{t-1}^I(i_t) \Big) F_{t-1}^J(j_{t-1}) \tag{S32}$$

Similarly, in the denominator, the sum over j_t may be brought in front of the final term, giving

$$F_{t-1}^J(j_{t-1}) \sum_{i_t=0}^{i_{t-1}} F_t^I(i_t) f_{t-1}^I(i_t) \sum_{j_t=0}^{\infty} f_{t-1}^J(j_t) \tag{S33}$$

$$= F_{t-1}^J(j_{t-1}) \sum_{i_t=0}^{i_{t-1}} F_t^I(i_t) f_{t-1}^I(i_t) \tag{S34}$$

since the infinite sum is equal to 1. The (Eq. S32) and denominator (Eq. S34) are equal, giving the logarithm an argument of 1 and making the overall term's value zero. This happens for all eight terms in the contextual STE expression (Eq S11), yielding a contextual STE that is exactly equal to zero. So, it is guaranteed that when one age group does not contribute infection to another, the contextual STE in that direction will be zero, as required.

To explore further characteristics of the contextual STE, consider the Poisson-type epidemiological model given in Eqs S18-S19. For three different sets of within- and between-group rates of transmission, we check how the contextual STE varies with reproduction number R and contextual case counts i_{t-1} and j_{t-1}.

To lay the groundwork, we seek an expression for the next-generation matrix $\boldsymbol{\lambda}$ in terms of the reproduction number R and some relative rates of within- and between-group transmission. Define the relative rate matrix

$$\boldsymbol{r} = \begin{bmatrix} r_{11} & r_{12} \\ r_{21} & r_{22} \end{bmatrix} \tag{S35}$$

where element r_{ij} is the relative rate at which an infected individual in class j infects individuals in class i. So, for example, if $r_{12} = 2r_{21}$, then the infection rate from group 2 to group 1 is double the infection rate from group 1 to group 2. Let ρ be the dominant eigenvalue of $\boldsymbol{r}$. The next-generation matrix is

$$\boldsymbol{\lambda} = \frac{R}{\rho} \boldsymbol{r}. \tag{S36}$$

This ensures that the dominant eigenvalue of $\boldsymbol{\lambda}$ is R, and that the relative magnitudes of the elements of $\boldsymbol{\lambda}$ match the relative magnitudes of the elements of $\boldsymbol{r}$. Note that using a constant multiple of the relative rate matrix $\boldsymbol{r}^* = c\boldsymbol{r}$ will still yield the same next-generation matrix $\boldsymbol{\lambda}$, since the dominant eigenvalue ρ^* of $\boldsymbol{r}^*$ will simply divide out the constant c again.

First, consider equal within- and between-group rates of transmission; that is,

$$\boldsymbol{r} = \begin{bmatrix} 1 & 1 \\ 1 & 1 \end{bmatrix}. \tag{S37}$$

This describes mean-field dynamics between the age groups. The group that dominates transmission should therefore be the group with the most cases, at least during the phase roughly prior to the epidemic peak (when $R_{\text{eff}} > 1$), since there are no intrinsic differences in transmission rates. That is, if there are (somehow) 100 infected children and just one infected adult, children will be responsible for the bulk of new cases, even though each child individually has exactly the same transmission potential as the infected adult. We calculate the contextual STE for reproduction number R between 0.6 and 1.5, which covers a range of possible reproduction numbers for influenza (see, for example, Smieszek *et al.* (2011) [3]). The number of cases in class I at time $t-1$, i_{t-1}, is held fixed at 25, and the number of cases in class J at time $t-1$, j_{t-1}, is allowed to vary from 5 to 50. Though these are small numbers of cases, they coincide with the weekly numbers of recorded ILI cases in some of the smaller age groups in the SDI-ILI dataset, especially in infants and the elderly (see Fig S13).

Fig S4 depicts $T^S_{I\to J,t-1} - T^S_{J\to I,t-1}$ for $R \in [0.6, 1.5]$ and $j_{t-1} \in \{5, \ldots, 50\}$. Fig S4 is generated by considering pairs of j_{t-1} and R, with j_{t-1} between 5 and 50 in steps of 5, and R between 0.6 and 1.5 in steps of 0.05. For each pair of j_{t-1} and R, the contextual STE (Eq S11) is calculated in both directions ($I \to J$ and $J \to I$), using the summation terms expressed as in Eq S30. Contours depict the difference between these contextual STEs. Note that i_{t-1}, j_{t-1}, $\boldsymbol{r}$, and R are sufficient to specify F^I, F^J, f^I, and f^J, using Eq S20–S21.

When $R > 1$, the age group with more cases transfers the most information. That is, when $j_{t-1} < i_{t-1} = 25$ (Fig S4, upper left quadrant), $T^S_{I\to J,t-1} > T^S_{J\to I,t-1}$, and when $j_{t-1} > i_{t-1} = 25$ (upper right quadrant), $T^S_{I\to J,t-1} < T^S_{J\to I,t-1}$. This matches with what one might expect from the epidemiological dynamics: when $R > 1$, the age group with more infected individuals causes the majority of new infections, and so has a higher contextual STE. When $R < 1$, interestingly, the reverse is true; the contextual STE is higher from the group with fewer cases. This is a spurious result arising from the STE not taking into account the underlying epidemic process. Clearly, epidemic decay is not ‘driven’ in the same way transmission is; decay is governed by individual recovery rates, and not by interactions between individuals. STE, however, searches simply for patterns in one process that predict patterns in another. During an epidemic's decay, one age group's dropping case counts may well anticipate drops in another group's case counts simply because the depletion of susceptibles in the first age group occurs earlier than in the second. The contextual STE identifies this relationship, and identifies the process with fewer cases as the one that is driving the decay. This suggests that caution is warranted when interpreting differences in STE; it must always be done with reference to the underlying epidemiological dynamics. In future work, it may be worthwhile to consider incorporating epidemiological intuition explicitly into the STE formulation; the simplest way to do this may be to restrict attention to only symbols that represent rises in amplitude. To summarise, when intrinsic within- and between-group rates of transmission are equal ($\boldsymbol{r}$ given by Eq S37), the relative number of cases at time $t-1$ dictates

which group transfers the most information to the other. When $R > 1$, the group with more cases at time $t-1$ transfers the most information; when $R < 1$, the group with fewer cases at time $t-1$ transfers the most information.

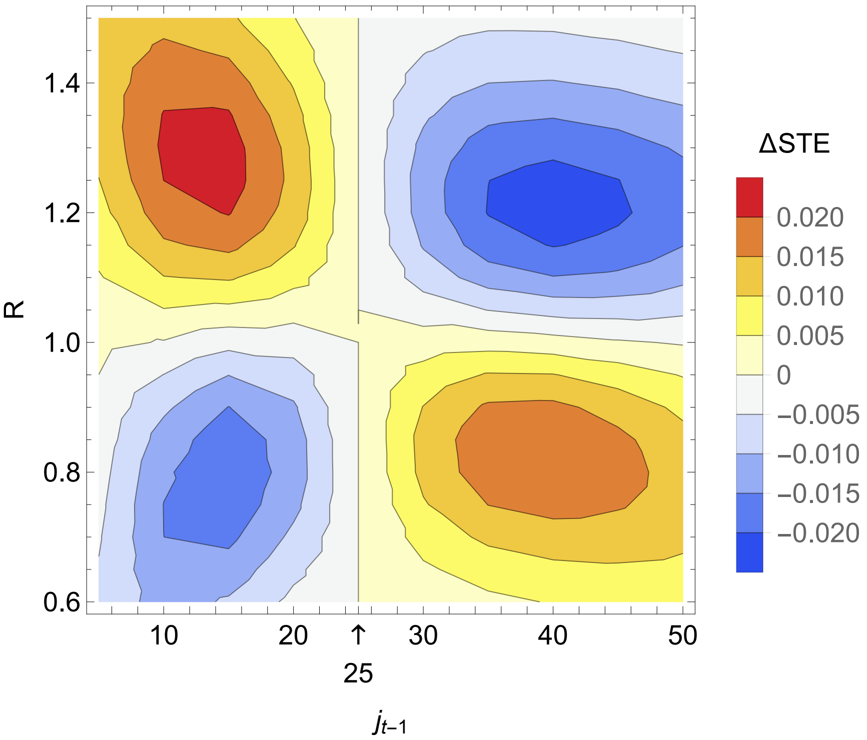

FIGURE S4. Difference in contextual STE, $\Delta\text{STE} = T^S_{I\to J,t-1} - T^S_{J\to I,t-1}$, for j_{t-1} between 5 and 50 and R between 0.6 and 1.5, with i_{t-1} fixed at 25. Units for the vertical scale are in bits. When $T^S_{I\to J,t-1} > T^S_{J\to I,t-1}$ (redder colours), there is evidence that process I drives process J more strongly than process J drives process I, and vice-versa. The relative rates of within- and between-group transmission are equal, as specified by the rate matrix $\boldsymbol{r}$ (Eq S37). The $I \to J$ contextual STE is higher when $i_{t-1} > j_{t-1}$ and $R > 1$ (upper left quadrant), and when $i_{t-1} < j_{t-1}$ and $R < 1$ (lower right quadrant). The $I \to J$ and $J \to I$ contextual STEs are approximately equal when $i_{t-1} = j_{t-1}$ and when $R = 1$.

Next, consider a case in which the within-group transmission rate for group I is twice that of group J, but all other infection rates are equal. That is,

$$\boldsymbol{r} = \begin{bmatrix} 2 & 1 \\ 1 & 1 \end{bmatrix}. \tag{S38}$$

In this scenario, sub-population I does not directly contribute to infection in sub-population J any more than J contributes to itself or to I. However, the growth rate in the number of infections in group I is higher than for group J, and so group I will eventually account for the bulk of transmission. As before, the contextual STE is calculated using the relative rate matrix, Eq S38, for $R \in [0.6, 1.5]$ and $j_{t-1} \in \{5, \ldots, 50\}$, with i_{t-1} fixed at 25. The contour plot in Fig S5 depicts $T^S_{I\to J,t-1} - T^S_{J\to I,t-1}$ under this scenario. The epidemiological intuition is supported; for

nearly the full range of parameters, $T^S_{I\to J,t-1} > T^S_{J\to I,t-1}$. For large j_{t-1} and $R > 1$ (upper right), and also for small j_{t-1} and $R < 1$ (lower left), the dominant transfer of information is reversed ($T^S_{I\to J,t-1} < T^S_{J\to I,t-1}$), but only by a small amount. The $I \to J$ contextual STE dominates most when j_{t-1} is small compared to i_{t-1} and when $R > 1$ – that is, when the transmission-dominant age group (I) has more cases and the outbreak is on the upswing.

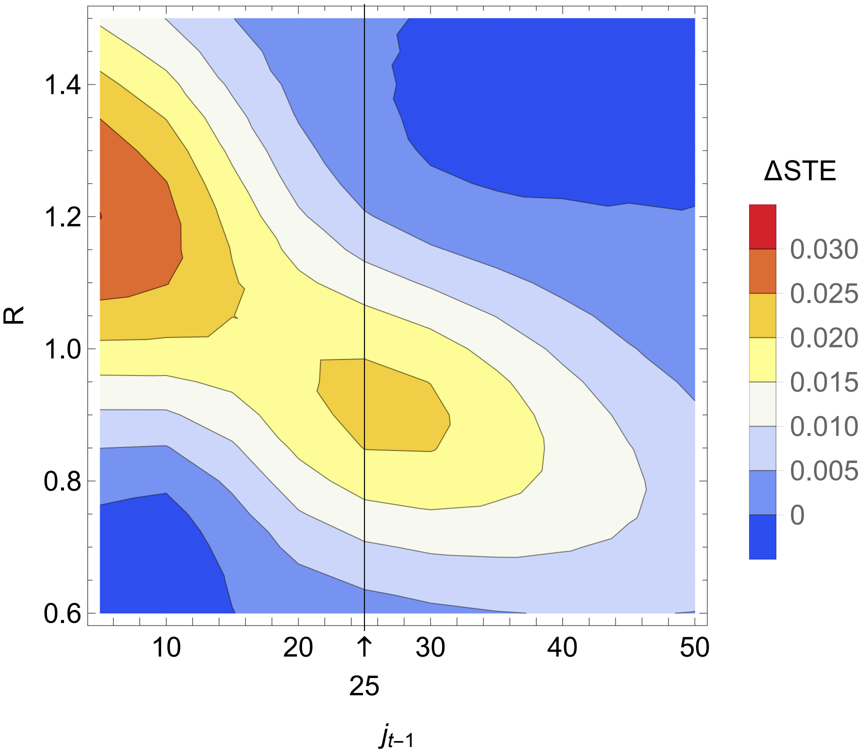

FIGURE S5. Difference in contextual STE, $\Delta\text{STE} = T^S_{I\to J,t-1} - T^S_{J\to I,t-1}$, for j_{t-1} between 5 and 50 and R between 0.6 and 1.5, with i_{t-1} fixed at 25. Units for the vertical scale are in bits. The within-group rate of infection for group I is double the other infection rates (see Eq S38). The $I \to J$ contextual STE dominates in most of the parameter space, especially when $j_{t-1} < i_{t-1} = 25$ and $R > 1$. The $J \to I$ contextual STE is slightly higher than the $I \to J$ contextual STE for large j_{t-1} and high R (upper right), and also for small j_{t-1} and low R (lower left), with ΔSTE reaching a minimum of -0.0032 bits at $j_{t-1} = 10$ and $R = 0.65$.

Finally, consider the case in which the within-group transmission rate for group I is quadruple the transmission rate from group J to group I, and the transmission rate from group I to group J is double the transmission rate from group J to group I. The within-group transmission rate for group J is equal to the transmission rate from group J to group I. That is,

$$\boldsymbol{r} = \begin{bmatrix} 4 & 1 \\ 2 & 1 \end{bmatrix}. \tag{S39}$$

This corresponds to strong driving of transmission from group I. Again, the contextual STE is calculated using the relative rate matrix Eq S39 for $R \in [0.6, 1.5]$ and $j_{t-1} \in \{5, \ldots, 50\}$, with i_{t-1} fixed at 25. The contour plot in Fig S6 depicts $T^S_{I\to J,t-1} - T^S_{J\to I,t-1}$ under this scenario. The

$I \to J$ contextual STE is dominant throughout the parameter space, and is most dominant when $i_{t-1} > j_{t-1}$ and $R > 1$. Here, the contextual STE correctly identifies that group I dominates transmission for the full range of epidemiologically-feasible parameter values.

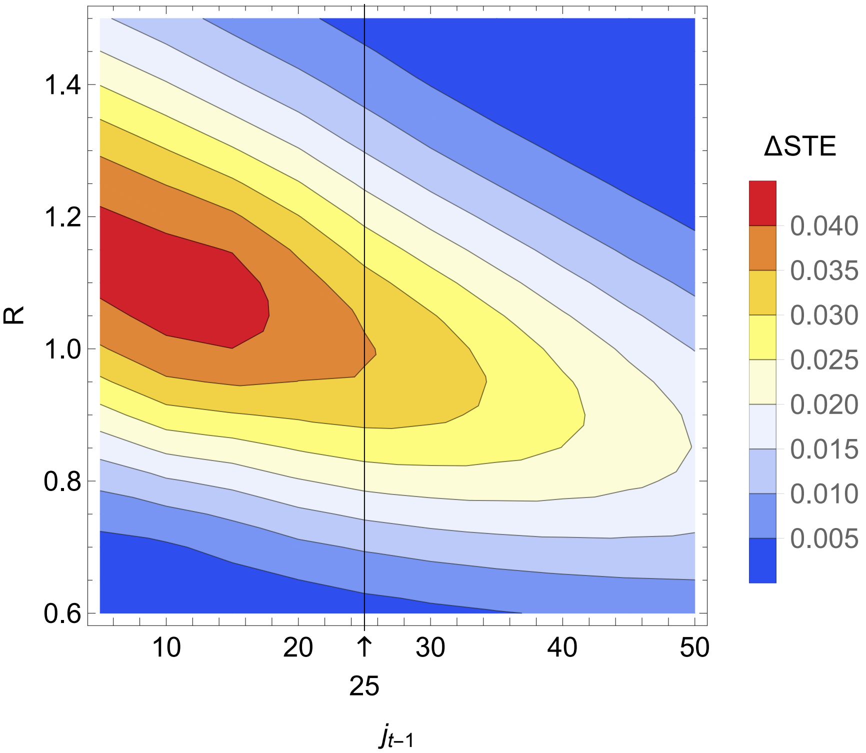

FIGURE S6. Difference in contextual STE, ΔSTE $= T^S_{I\to J,t-1} - T^S_{J\to I,t-1}$, for j_{t-1} between 5 and 50 and R between 0.6 and 1.5, with i_{t-1} fixed at 25. Units for the vertical scale are in bits. The within-group rate of infection for group I is quadruple the within-group rate of infection for group J, and the infection rate from group I to group J is double that from group J to group I (see Eq S39). The $I \to J$ contextual STE dominates throughout of the parameter space (ΔSTE > 0 everywhere), especially when $j_{t-1} < i_{t-1} = 25$ and $R > 1$.

3. SIMULATIONS

3.1. **The stochastic SIR model.** The rate of transmission $\beta_{i,j}$ from class j to class i may be calculated from the relative rate matrix $\boldsymbol{r}$, the basic reproduction number R_0, the recovery rate γ, the class-specific population sizes N_1 and N_2, and the total population size $N = N_1 + N_2$:

$$\boldsymbol{\beta} = \begin{bmatrix} \beta_{11} & \beta_{12} \\ \beta_{21} & \beta_{22} \end{bmatrix} = \frac{R_0\gamma}{\rho} \begin{bmatrix} N/N_1 & 0 \\ 0 & N/N_2 \end{bmatrix} \cdot \boldsymbol{r} \tag{S40}$$

Consider two age groups I and J, which might represent children and adults, respectively. For each age group, let $X_I(t)$ and $X_J(t)$ be the number of susceptible individuals, $Y_I(t)$ and $Y_J(t)$ be the number of infected individuals, and $Z_I(t)$ and $Z_J(t)$ be the number of recovered individuals at (continuous) time t. Let N be the total population size, equal to the total number of individuals in all age classes and disease states. N remains constant throughout the simulation; there are no births and no deaths. The population sizes of each age group, N_I and N_J, also remain constant throughout the simulation; individuals may not transition between the two age groups on the time scale of this epidemic. Individuals transition from susceptible to infected to recovered according to the rates given in Table S1. The parameter β_{mn} specifies the transmission rate from age group n to age group m. The recovery rate γ is assumed constant across age groups.

TABLE S1. Infection rates for the two-age-class SIR model

Transition	Rate
$X_I \to Y_I$	$\beta_{11}X_IY_I/N + \beta_{12}X_IY_J/N$
$X_J \to Y_J$	$\beta_{21}X_JY_I/N + \beta_{22}X_JY_J/N$
$Y_I \to Z_I$	γY_I
$Y_J \to Z_J$	γY_J

The next-generation matrix for this model is

$$NGM = \frac{1}{\gamma} \begin{bmatrix} \beta_{11}p_I & \beta_{12}p_I \\ \beta_{21}p_J & \beta_{22}p_J \end{bmatrix} \tag{S41}$$

where $p_I = N_I/N$ and $p_J = N_J/N$. The within- and between-group transmission dynamics therefore depend on the infection rates β_{mn} and the relative population sizes N_I and N_J.

Let $\boldsymbol{r}$ be a matrix in which the i,j^{th} entry gives the relative rate of infection from age class j to age class i. For a given basic reproduction number R_0 and recovery rate γ, the transmission rates can be calculated:

$$\boldsymbol{\beta} = \begin{bmatrix} \beta_{11} & \beta_{12} \\ \beta_{21} & \beta_{22} \end{bmatrix} = \frac{R_0\gamma}{\rho}\text{diag}(1/p_I, 1/p_J) \cdot \boldsymbol{r} \tag{S42}$$

where ρ is the dominant eigenvalue of $\boldsymbol{r}$. This yields a next-generation matrix with dominant eigenvalue equal to R_0, in which the proportional differences between the terms match those of the rate matrix $\boldsymbol{r}$.

Table S2 lists the parameter values used for the individual-based SIR simulations. The basic reproduction number R_0 is fixed at 1.5, which is consistent with estimated values of R_0 for 2009

pandemic influenza [4, 5]. The average time to recovery $1/\gamma$ is 7 days, which is in line with estimates of the infectious period for influenza [6]. The population sizes N_I and N_J are small enough to ensure clearly stochastic dynamics (the dynamics become nearly deterministic as the population sizes increase), and are in line with the population sizes of the smaller age groups in the SDI-ILI dataset.

TABLE S2. Parameter values for the two-age-class SIR model

Parameter	Value	Description	Units
R_0	1.5	Basic reproduction number	people
γ	1/7	Recovery rate	1/day
N_I	500	Number of individuals in age group I	people
N_J	500	Number of individuals in age group J	people

Simulations are implemented using the Gillespie algorithm, starting with one infected individual placed uniform-randomly in either age group. Since R_0 is relatively low, there is a high chance of early epidemic die-out, so only outbreaks that trigger at least 100 total cases are recorded. Once an outbreak is simulated, infections are binned into week-long intervals. Poisson noise is added to each bin at a rate of 0.5% of the population size to simulate background non-influenza ILI. This rate is just below the 0.6% ILI ratio cutoff used in Gog *et al.* (2014) [7] to define out-of-season, low ILI activity weeks. Fig S7 depicts five simulated epidemic time series from this model.

3.2. **The Poisson model.** For the Poisson model, let the number of infected individuals in age class i at (discrete) time t follow

$$Y_{i,t} \sim \text{Poisson}(\sum_j \lambda_{ij,t} Y_{j,t-1}) \tag{S43}$$

where $Y_{i,t}$ is the number of infected individuals of class i at time t, and $\lambda_{ij,t}$ is the infection rate from class j to class i at time t. The length of the time steps is assumed to coincide with the generation interval for the disease, which we set throughout at 3.5 days. Unlike in the stochastic SIR model, the infection rates $\lambda_{ij,t}$ are allowed to vary in time.

For a given relative reproduction matrix $\boldsymbol{r}$ where element r_{ij} denotes the relative reproduction ratio from group j to group i, and a given effective reproduction number R_t, the effective NGM may be expressed as

$$NGM_t = \boldsymbol{\lambda}_t = \frac{R_t}{\rho}\boldsymbol{r} \tag{S44}$$

where ρ is the dominant eigenvalue of the matrix $\boldsymbol{r}$. As before, this ensures that the dominant eigenvalue of the effective NGM is equal to the effective reproduction number R_t, and that the relative magnitudes of the elements of the effective NGM match the relative magnitudes of the elements of $\boldsymbol{r}$.

Epidemics are simulated by placing a single initial infected individual uniform-randomly into one of the age groups, and then simulating the numbers of infected individuals in each group for subsequent weeks according to draws from the Poisson distribution specified by Eq. S43. For the first eight time steps (28 days), R is fixed at 1.5. After the eighth time step, R is decreased

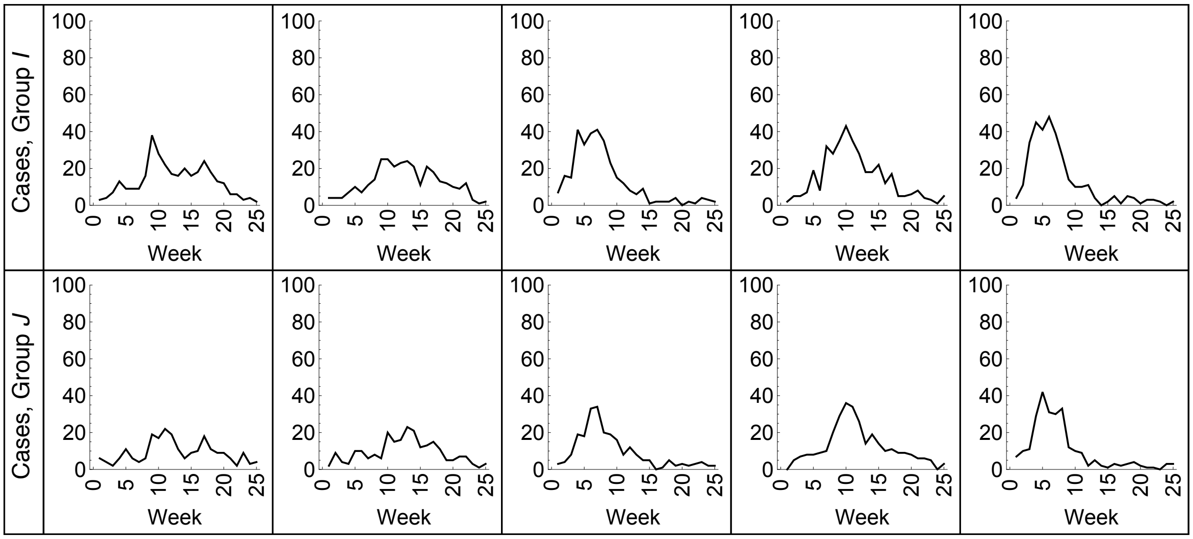

FIGURE S7. Five simulations from the two-age-class stochastic SIR model, implemented using the Gillespie algorithm. Each column depicts the output from a single epidemic simulation, separated into case counts for group I (top) and group J (bottom). Vertical axes have units of case counts, and horizontal axes have units of weeks. Transition rates are given in Table S1, with parameter values in Table S2. The transmission rates are specified by the relative rate matrix Eq. 4 (main text) with $z_b = 1$, for which age group I (upper row) has quadruple the within-group transmission rate as group J, and for which the $I \to J$ transmission rate is twice the $J \to I$ transmission rate.

0

to 0.8 for the rest of the epidemic. This yields outbreaks of similar size as the ones simulated using the stochastic SIR model (compare Figs S7 and S8). Only outbreaks in which at least 100 people become infected are recorded. Five simulations from a two-age-class Poisson-type model are depicted in Fig S8.

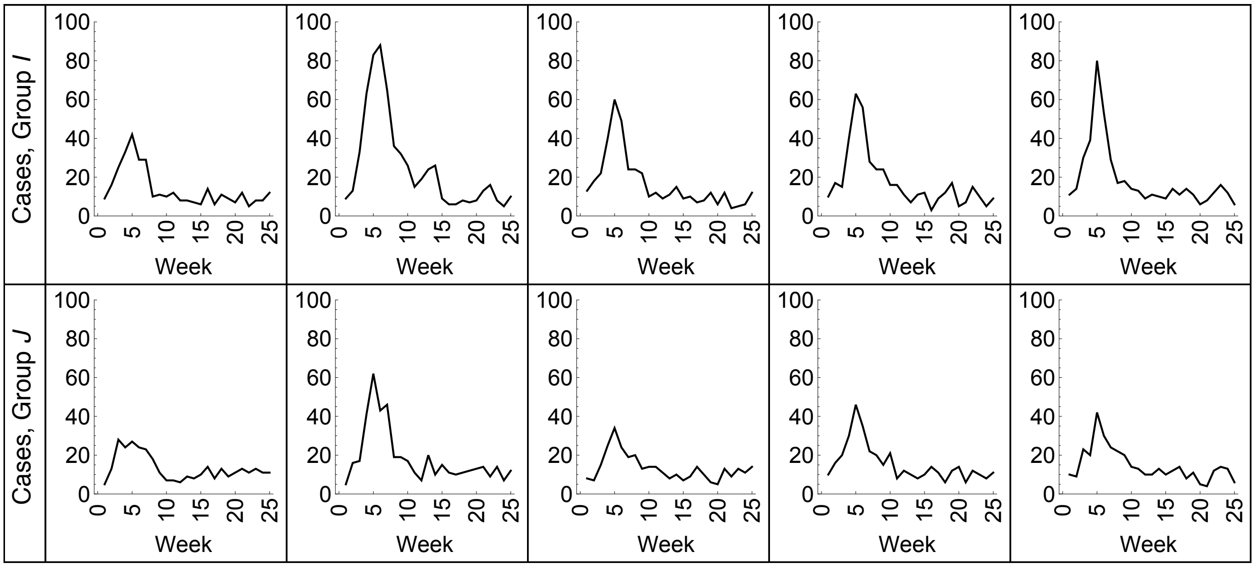

FIGURE S8. Five simulations from the two-age-class Poisson-type simulation model, Eq. S43. Each column depicts the output for a single epidemic simulation, separated into case counts from group I (top) and group J (bottom). The reproduction number R is 1.5 for the first eight weeks of the outbreak, and then drops to 0.8 for the rest of the epidemic. The transmission rates are specified by the relative rate matrix Eq. 4 (main text) with $z_b = 1$, for which age group I (upper row) has quadruple the within-group transmission rate as group J, and for which the $I \to J$ transmission rate is twice the $J \to I$ transmission rate.

4. EFFECT OF VARIABLE REPORTING RATE ON STE USING SIMULATION

The ability to detect differences in transmission strength between demographic groups with STE depends on reporting rate. In general, the differences are more detectible with higher reporting rates. Figure S9 depicts the relationship between reporting rate c (see Eq. 2) and STE using Poisson-type epidemic simulations strongly driven by Group 2 (relative rate matrix given by Eq. 5).

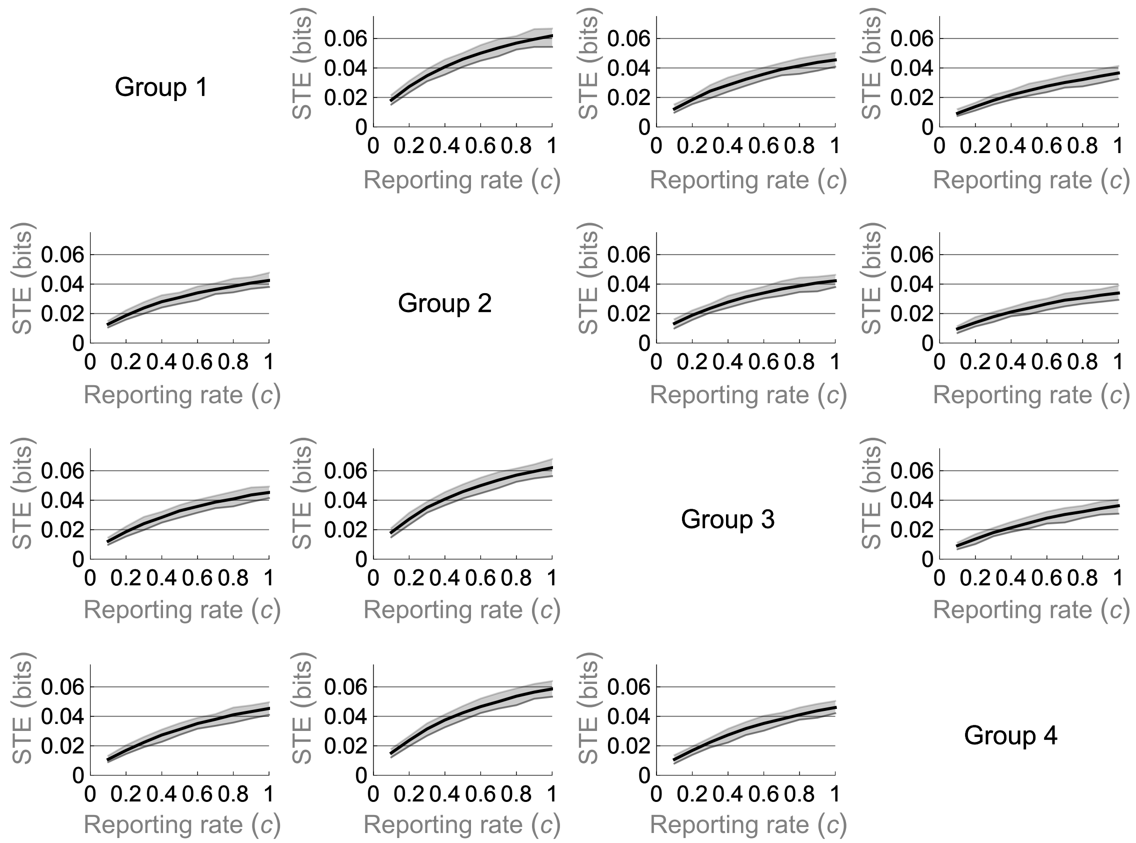

FIGURE S9. Mean pairwise STE values (solid lines) with 95% confidence intervals (shaded bands) for epidemics strongly driven by Group 2 under a range of reporting rates c. The curves are produced by simulating 100 ensembles of 800 epidemics each from the Poisson model for each value of c between 0.1 and 1 in steps of 0.1, and then calculating the between-group STE for each ensemble. The reporting rate c_i (see Eq. 2) is varied uniformly across all age groups i. The relative reproduction matrix that specifies within- and between-group transmission rates is given by Eq. 5. The plot in row i and column j depicts the STE from group j to group i.

5. Effect of variable population size on STE using simulations

As discussed in Section S2.1, the relative transmissive importance of different age groups as measured by the contextual STE can depend on the relative population sizes of the age groups: the contextual STE tends to be higher for groups with larger population sizes. According to epidemic simulations, this tends to hold true for the full STE as well. Fig. S10 depicts the STE between two groups for epidemics simulated using the SIR model (see Section S3.1) for the relative reproduction matrices considered in Section 3.1 of the main text. The total population size remains fixed at 1,000 individuals, but for the first row of plots, group 1 (blue) has 250 individuals and group 2 (black) has 750; for the second row, group 1 has 500 individuals and group 2 has 500; and for the third row, group 1 has 750 individuals and group 2 has 250. For the left-hand column of plots, the epidemiological coupling between the groups ranges from none to fully symmetric. For the right-hand column of plots, the epidemiological coupling between the groups ranges from fully symmetric to strongly driven by group 1. When the population size of group 1 is smaller than that of group 2 (Fig. S10 top row), the group 2 $\to$ 1 STE (black) dominates until $z_b \approx 0.4$, when group 2 has substantially stronger transmission to group 1 than *vice-versa*. Conversely, when the population size of group 1 is larger than that of group 2 (Fig. S10 bottom row), the group 1 $\to$ 2 STE (blue) dominates throughout.

For the 2009 A/H1N1pdm influenza pandemic, we find that school-aged children dominated transmission of the outbreak, according to the pairwise estimated STE values (see Section 3.4, main text). In the SDI-ILI dataset, the age groups are partitioned such that these younger age groups span fewer years, and therefore tend to contain fewer individuals, than the older age groups. The fact that the pairwise STE values from these younger age groups to the other age groups tend to be high is good evidence that these younger age groups were in fact strong drivers of transmission during the 2009 A/H1N1pdm influenza pandemic. If the observed STE patterns were due to differences in the population sizes of the age groups, we would expect the patterns to be the opposite of what we observe, with the older (larger) age groups appearing to drive transmission.

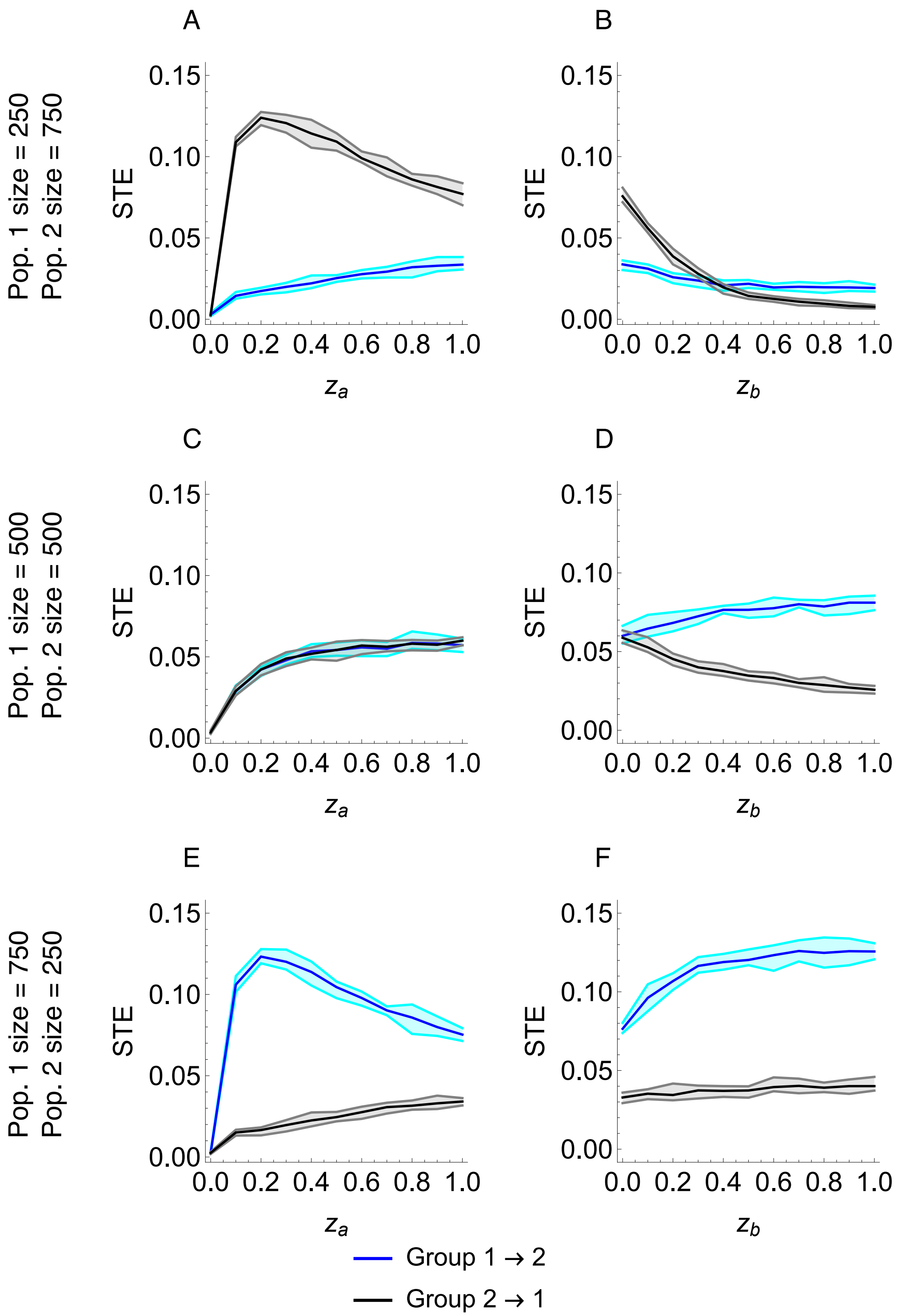

FIGURE S10. Mean (95% CI) Group 1→2 (blue) and Group 2→1 (black) STE values as the coupling between the groups ranges from none to fully symmetric (left-hand column), and from fully symmetric to strongly driven by Group 1 (right-hand column) for different relative population sizes between the two age groups. The curves are produced by simulating 100 ensembles of 800 epidemics each from the stochastic SIR model for each value of z_a and z_b between 0 and 1 in steps of 0.1 (see Eqs 3 and 4), and then calculating the between-group STE for each ensemble.

6. Relationship between STE and optimal vaccination

We do not currently advocate using STE on its own to guide vaccination strategies. A full description of the contexts in which STE accurately identifies which population groups to vaccinate in order to maximally reduce the final size of an outbreak lies beyond the scope of this paper. Here, we present preliminary data showing that, under the modelling assumptions presented in this paper, vaccinating groups with higher STE tends to more effectively reduce the mean final size of simulated outbreaks.

We generated 100 random 4×4 relative reproduction matrices by choosing each element according to a *uniform*(0,1) distribution. For each relative reproduction matrix, we used the Poisson outbreak model to generate a single ensemble of 800 outbreaks. For each ensemble, we calculated the pairwise STE between age groups. Then, for each relative reproduction matrix in the original set, we simulated 500 more outbreaks with group 1 vaccinated, 500 outbreaks with group 2 vaccinated, and so on. To simulate vaccination, we simply did not allow members of the vaccinated group to become infected. From each vaccination scenario and each relative reproduction matrix, we calculated the mean final outbreak size using these 500 simulations. This allowed us to link the total STE 'from' a given age group (to all other age groups) to the mean final outbreak size that would result if that age group were vaccinated, given a relative reproduction matrix. Fig. S11 depicts this relationship. The decreasing trend indicates that greater reductions in mean epidemic size result when age groups with higher total STE 'from' are vaccinated.

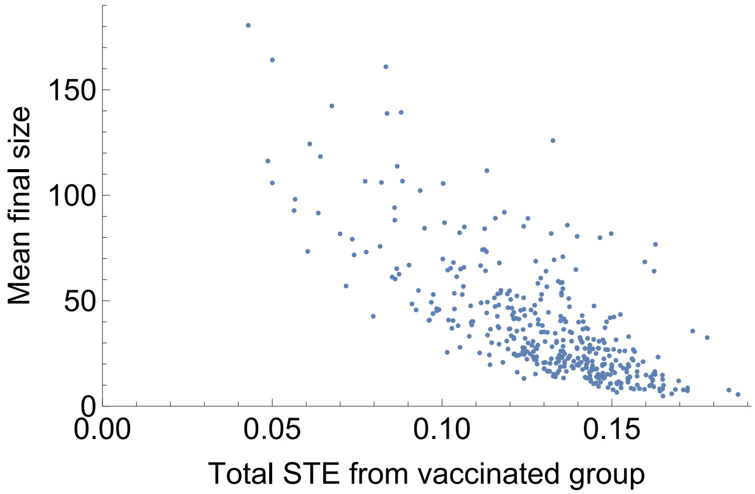

FIGURE S11. Mean final outbreak size with one age group vaccinated as a function of the total STE 'from' that age group to all other age groups. The mean sizes were generated from 500 independent Poisson-type outbreak simulations with four age groups.

7. ROBUSTNESS TO VARIATION IN REPORTING RATES

To test whether the differences in pairwise STE observed from the SDI-ILI data could be fully explained by differences in reporting rates between age groups, pre-reporting counts of influenza-like illness can be roughly inferred from the observed ILI counts, and the STE may be re-calculated. If the STE trends persist, then they are likely robust to reporting uncertainty. To accomplish this, it is necessary to first demonstrate that the STE differences observed in the ILI data still hold when estimated using ILI counts rather than the ILI ratios. Fig. S12 depicts the pairwise STE between each age group calculated using the raw ILI counts rather than the ILI ratios (that is, without normalising by the number of physician visits in each ZIP in each week). The same patterns hold, with school-aged children emerging as the primary contributors of information to most other age groups' time series.

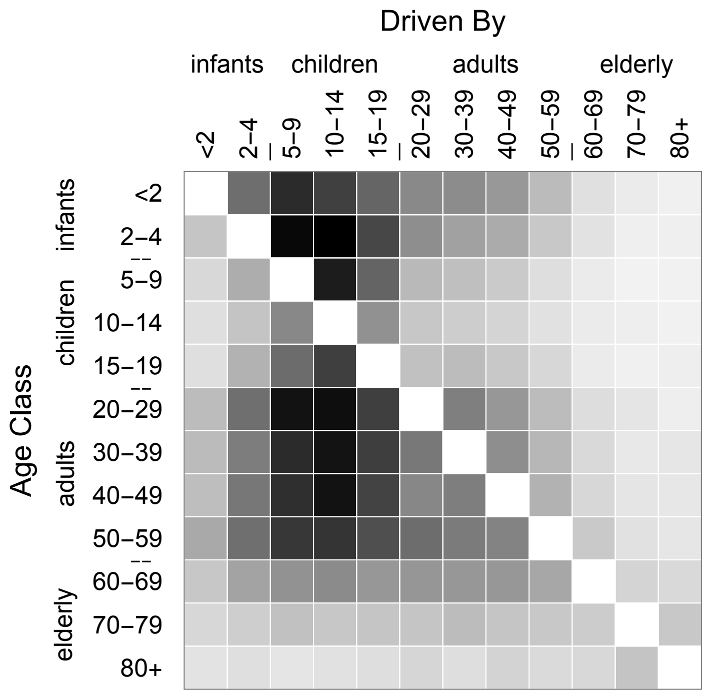

FIGURE S12. Pairwise STEs between age groups, using ILI counts rather than ILI ratios. Darker boxes correspond to higher values. The values are almost identical to the ones obtained using ILI ratios (see Fig 4, main text).

Next, it is possible to reconstruct a distribution of possible true case counts from the observed case counts, given a particular reporting rate. First, for a given ZIP, we assume that the observed ILI count $y_{i,t}^{obs}$ in age band i in week t represents a binomial sample from $y_{i,t}$ total ILI cases in that week. That is,

$$y_{i,t} \sim Binomial(y_{i,t}, c_i) \qquad \text{(S45)}$$

where c_i is the reporting rate for age band i. Given the number of observed cases $y_{i,t}^{obs}$ and reporting rate c_i, the normalised likelihood for the true number of cases in week t is

$$L(y_{i,t}; y_{i,t}^{obs}, c_i) = \binom{y_{i,t}}{y_{i,t}^{obs}} c_i^{y_{i,t}^{obs}+1}(1-c_i)^{y_{i,t}-y_{i,t}^{obs}} \tag{S46}$$

which satisfies

$$\sum_{y_{i,t}=0}^{\infty} L(y_{i,t}; y_{i,t}^{obs}, c_i) = 1. \tag{S47}$$

The normalised likelihood for $y_{i,t}$, Eq. S46, may be interpreted as a probability distribution, from which possible 'true' numbers of cases in week t can be drawn.

To test the robustness of the STE results to reporting uncertainty, possible 'true' case counts are drawn using Eq. S46 for each week and each age group in each location, and the pairwise STE is re-calculated. This is repeated 100 times. The reporting rate c is assumed to be 60% for ages 0–19 years, and 40% for ages 20 and above, following the ILI reporting rate estimates from the 2009 A/H1N1pdm pandemic in the US reported by Biggerstaff *et al.* (2012) [8]. Fig S13 depicts four reconstructed full case counts (grey) from four age-group time series (rows) in four different locations (columns). Fig S14 depicts the mean pairwise within-ZIP STE between age groups using the reconstructed case counts. The pattern of elevated STE from children to infants through adults persists.

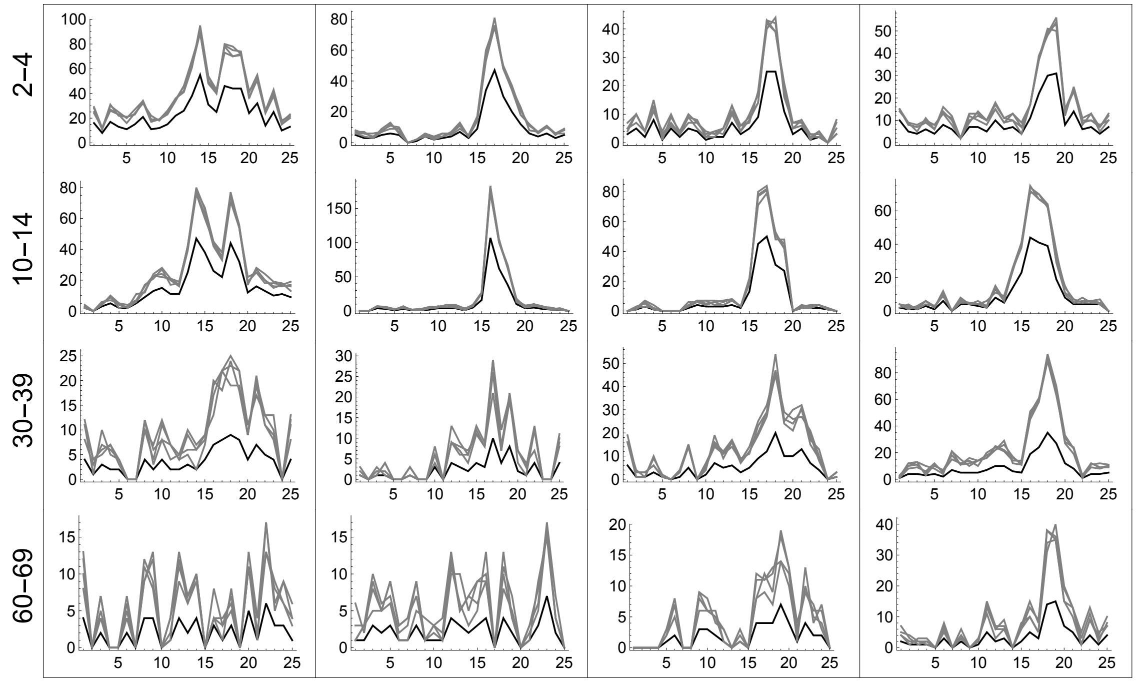

FIGURE S13. Observed ILI counts (black) with four binomial reconstructions (grey) of the 'true' numbers of counts for four age groups in four ZIPs. It is assumed that children (under 20 years) have 60% reporting rates and adults (20+) have 40% reporting rates, following [8].

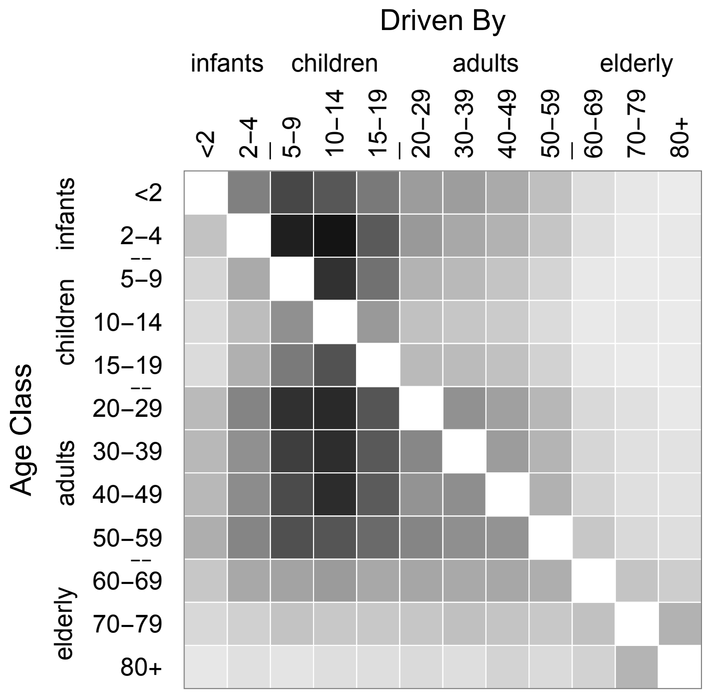

FIGURE S14. Mean pairwise within-ZIP STE values estimated from 100 reconstructed ILI case-count time series, assuming a 60% reporting rate in infants and children and a 40% reporting rate in adults and elderly. Darker boxes correspond to higher values. School-aged children still transfer the most information to the other age groups, consistent with all of the previous results.

8. Symbolic transfer entropy in the post-pandemic period

In the months following the major autumn wave of the 2009 A/H1N1pdm influenza pandemic outbreak in the United States, influenza transmission declined substantially [4]. We expect ILI in this period to reflect sporadic respiratory illness, rather than the strong waves of infection that characterised the pandemic. Correspondingly, we expect to see little evidence of age structure in the pairwise STE calculations.

We calculated the pairwise STE between the 12 age groups represented in the SDI-ILI dataset during the 25 weeks between 10 January 2010 and 27 June 2010. These values are depicted in Fig. S15. Darker shades represent higher STE, and the boxes are shaded using the same scale as in Fig. 4 (main text). There is perhaps slight evidence of elevated transmission between similarly-aged groups (darker boxes along the diagonal). As expected, however, there is comparatively little evidence of age structure in ILI transmission during these post-pandemic months.

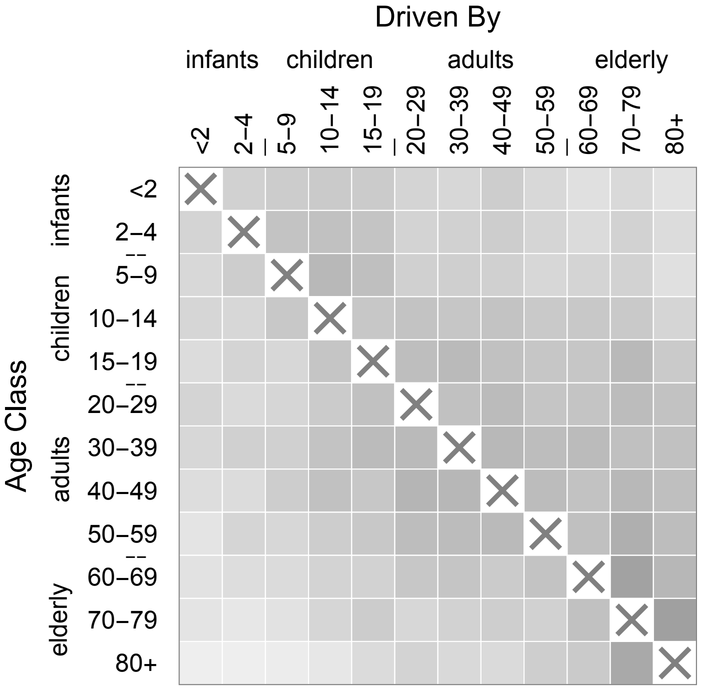

FIGURE S15. Mean pairwise STE values between the 12 groups represented in the SDI-ILI dataset during the six months following the autumn 2009 A/H1N1pdm pandemic influenza outbreak. A box in row i and column j corresponds to the STE from group j to group i, where darker shades corresponds to higher STE. Comparing with Fig. 4, there is little evidence of age structure in ILI transmission during this post-pandemic period.

9. AGE STRUCTURE IN OTHER INFLUENZA SEASONS, 2003–2008

In addition to the 2009 A/H1N1pdm influenza pandemic, we also calculated the pairwise STE between age groups using the SDI-ILI data for six previous influenza seasons, from 2003–2008. These values are depicted in Fig. S16. There is evidence of age structure in all six seasons, with elevated transmission between similarly-aged groups (darker squares along the diagonal) and some evidence of elevated transmission from children (darker squares in the lower-left quadrant) throughout. The variability in pairwise STE changes from season to season, however; 2004-05 was the least variable season and 2008-09 the most variable, as measured by the coefficient of variation (the standard deviation of the pairwise STE values divided by the mean pairwise STE). The maximum pairwise seasonal STE also varies from season to season: the highest maximum pairwise STE is observed in 2003-04 (0.067), and the lowest in 2008-09 (0.050). The 2009 A/H1N1pdm pandemic has a higher maximum pairwise STE and a greater coefficient of variation than any of the seasonal outbreaks, which provides evidence that the age structure of transmission during the pandemic was both stronger and more heterogeneous than for the preceding seasonal outbreaks. These statistics are given for all seasons in Table S3.

TABLE S3. Mean and variation in pairwise STE values by season

Season	Week range	Max pairwise STE	Coeff. of Variation
2003-04	2 Nov 2003 – 18 Apr 2004	0.067	0.42
2004-05	7 Nov 2004 – 24 Apr 2005	0.052	0.37
2005-06	6 Nov 2005 – 23 Apr 2006	0.058	0.39
2006-07	5 Nov 2006 – 22 Apr 2007	0.054	0.41
2007-08	4 Nov 2007 – 20 Apr 2008	0.057	0.43
2008-09	2 Nov 2008 – 19 Apr 2009	0.050	0.44
2009 pandemic	12 Jul 2009 – 27 Dec 2009	0.084	0.71

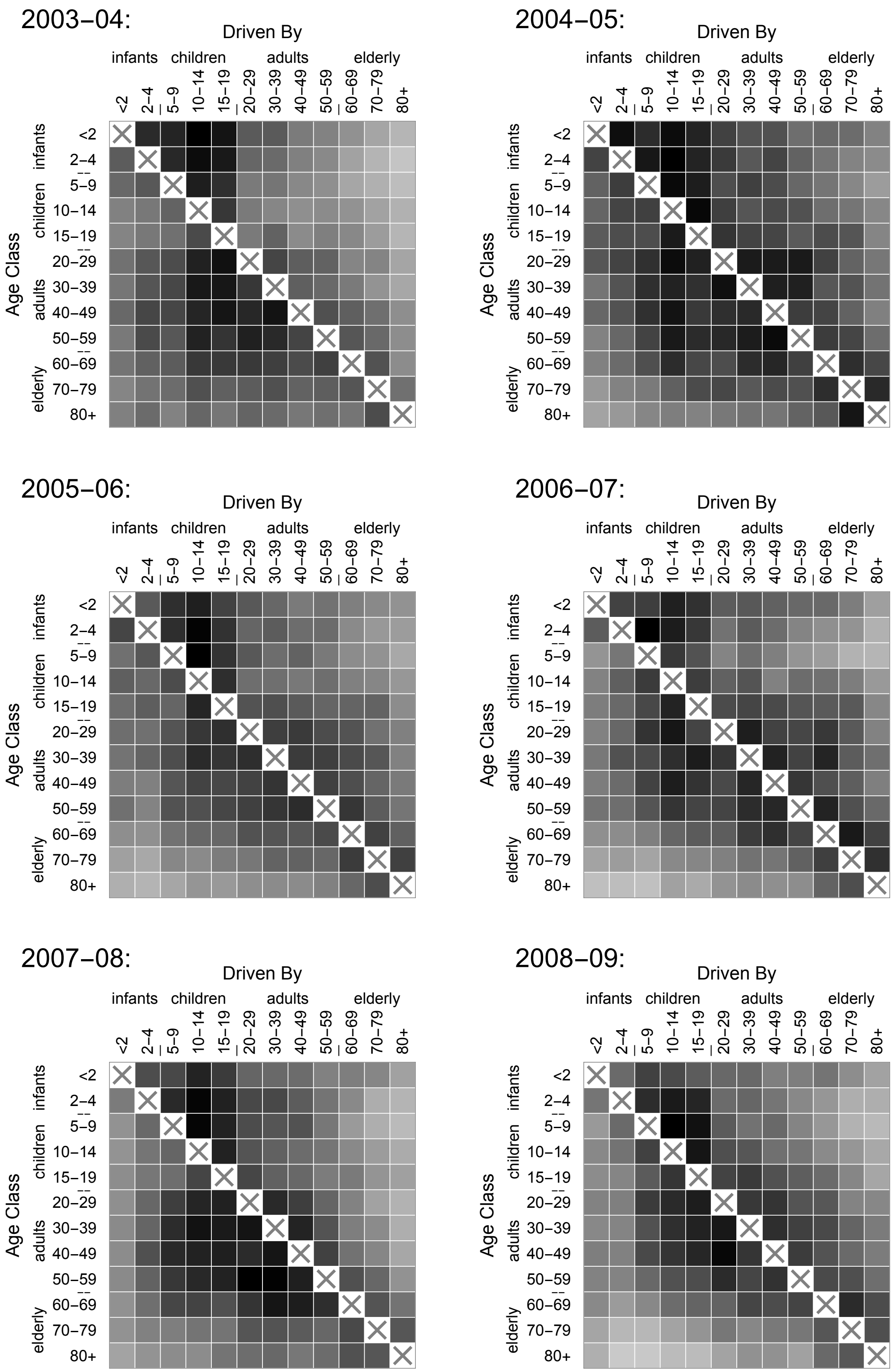

FIGURE S16. Mean pairwise STE values between the 12 groups represented in the SDI-ILI dataset during the six seasonal influenza outbreaks preceding the 2009 A/H1N1pdm pandemic. A box in row i and column j corresponds to the STE from group j to group i, where darker shades corresponds to higher STE. The shades are normalized within each year so that the highest pairwise STE in that year is black, and an STE of 0 is white.

10. Maximum-information symbols

It is possible to identify which symbols carry the most information in a given stochastic process. For example, if one time series has the symbol A at time t, it is possible to identify whether this provides more information about which symbol another time series will display at time $t+1$ than if the first time series had a different symbol at time t. This is done by separating the STE calculation in Eq. S7 into parts. The amount of information that an A in process J at time $t-1$ provides about whether the symbol in process I is a B at time t is

$$T^{S,AB}_{J\to I} = \sum_{\hat{i}_t} p(\hat{i}_{t+1} = B, \hat{i}_t, \hat{j}_t = A) \log\Big(\frac{p(\hat{i}_{t+1} = B|\hat{i}_t, \hat{j}_t = A)}{p(\hat{i}_{t+1} = B|\hat{i}_t)}\Big). \tag{S48}$$

The amount of information that each symbol contributes to every other symbol can thus be calculated. Using the SDI-ILI data, the symbol-specific STE is calculated from the three school-aged-children age groups (5-19, 10-14, and 15-19 years) to the age-aggregated time series in their same ZIP. The values are depicted in Fig S17. A symbol A (steadily increasing) in the child time series provides a lot of information that there will be an A in the overall time series at the next time step, and similarly for the symbol F (steadily decreasing). Symbols that end on an increase/decrease tend to predict symbols that also end on an increase/decrease, though there are exceptions. Overall, this suggests that the most informative symbols are strong increases and strong decreases (symbols A and F), which matches with the intuition that repeated increases and decreases in ILI tend to correspond to takeoffs of infection or declines in infection – epidemiologically relevant events that should affect the dynamics in other time series and thus transmit information to them. Other symbols are more likely to arise through stochastic noise, and so there is lower transfer of information between them.

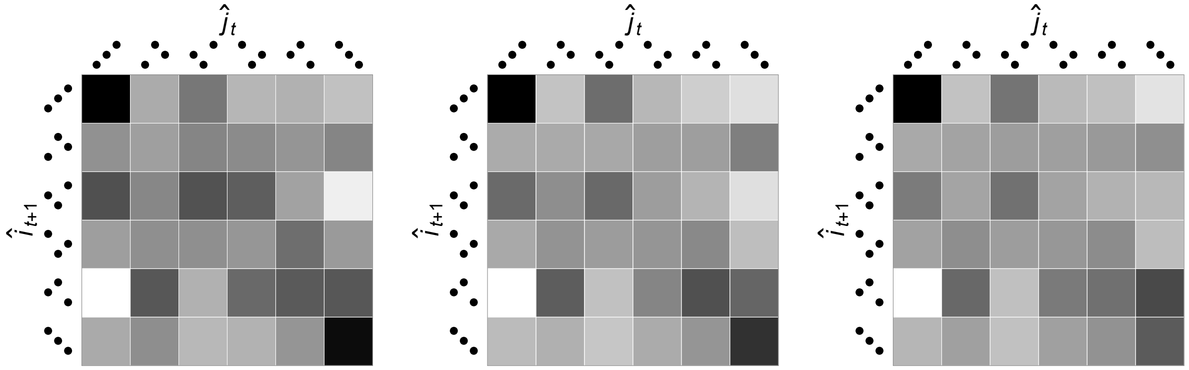

Figure S17. Information that each symbol $\hat{j}_t$ in the child time series carries about the next symbol $\hat{i}_{t+1}$ the age-aggregated time series, for each group of school-aged children (ages 5-9, 10-14, and 15-19, from left to right). The more information the symbol in the child time series carries about the symbol in the age-aggregated time series, the darker the corresponding square. The steadily-increasing and steadily-decreasing symbols (A and F) carry the most information, and tend to predict the same symbol in the age-aggregated time series.

11. An alternate symbolization

Sometimes, distinguishing between similar symbols — such as the 'peak' symbols B and E, or 'trough' symbols C and D, for $m = 3$ — may not be especially important for calculating information transfer. On the other hand, reducing the symbol space, say from 6 symbols down to 4, could be computationally advantageous: with fewer possible transitions between symbols, it will be easier to estimate the remaining transition probabilities using fewer time series replicates. Here, we test whether an abridged version of the $m = 3$ symbolization, in which symbols B and E now constitute a single symbol and symbols C and D constitute another single symbol, such that the relative ordering of the first and last points in each triplet is ignored, is equally capable of identifying asymmetric transmissive relationships between epidemiological groups.

We first used SIR-type stochastic epidemic simulations as described in the main text, Section 3, to test how the new symbolization behaves with varying between-group transmission strengths, encoded by varying z_a and z_b. The results are depicted in Fig. S18, which may be compared with Fig. 1 (main text). Though the absolute value of the STE in the four-symbol case is smaller than in the six-symbol case (intuitively, this is because there is simply less potential information encoded by a four-letter alphabet than by a six-letter one), the results are qualitatively the same. The STE increases steadily as the symmetric coupling between the groups increases (Fig. S18A), and then quickly diverges as the group $1 \to 2$ transmission begins to dominate the group $2 \to 1$ transmission (Fig. S18B).

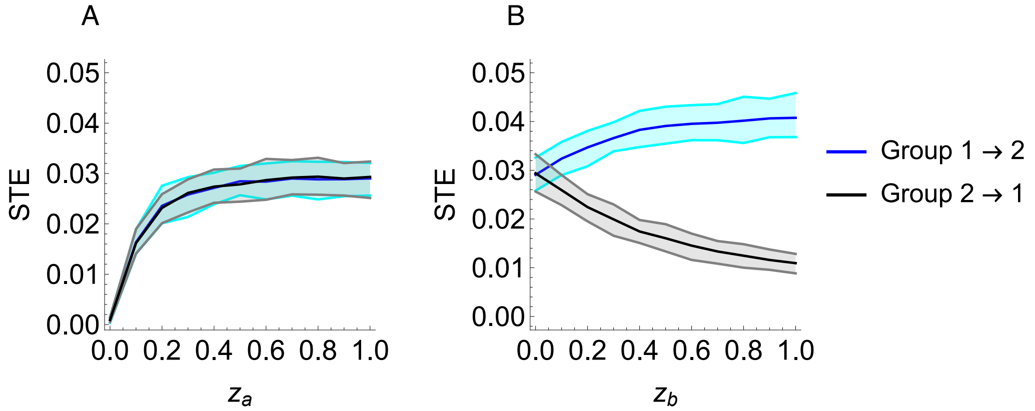

Figure S18. Mean (95% CI) Group 1→2 (blue) and Group 2→1 (black) STE values as the coupling between the two groups ranges from none to fully symmetric (A), and from fully symmetric to strongly driven by Group 1 (B). The curves are produced by simulating 100 ensembles of 800 epidemics each from the stochastic SIR model for each value of z_a and z_b between 0 and 1 in steps of 0.1, and then calculating the between-group STE for each ensemble. The epidemic time series are symbolised using the reduced four-symbol alphabet, collapsing symbols B and E as well as symbols C and D from the standard $m = 3$ symbolization. The relative reproduction matrices that capture these two coupling scenarios are given in Eqs 3 and 4 (main text).

We also used the reduced four-symbol alphabet to calculate the pairwise STE between age groups during the 2009 A/H1N1pdm influenza pandemic using the SDI-ILI data, as in Section 3.4

(main text). Qualitatively, the results are virtually indistinguishable (compare with Fig. 4, main text): there is evidence of relatively strong transmission from children aged 5–19 to ages 0–59. Again, the raw values of the STE using the four-symbol alphabet tend to be somewhat smaller than when using the six-symbol alphabet (max STE 0.051 vs. 0.084; compare Eq. S52 and Eq. S53), but this does not affect the general conclusion that school-aged children were likely the primary drivers of transmission during the 2009 A/H1N1pdm influenza pandemic in the US.

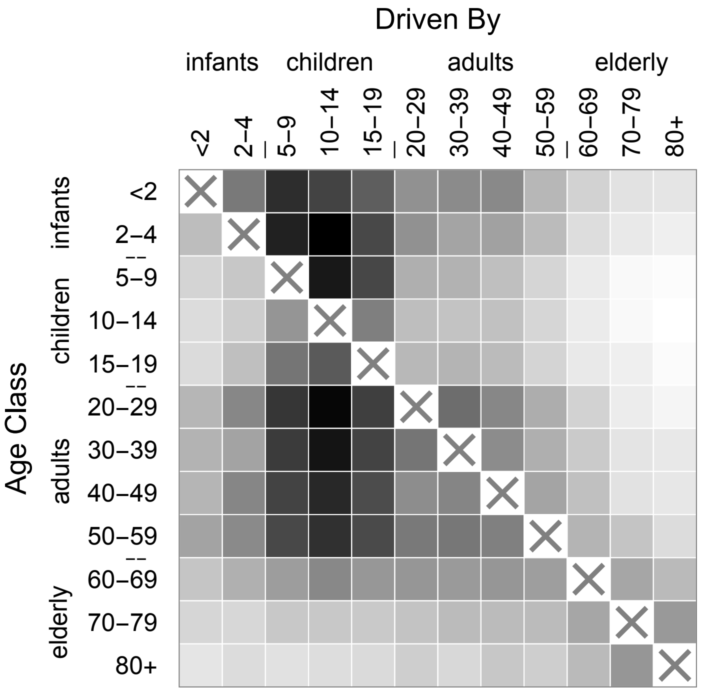

FIGURE S19. Mean pairwise STE values between the 12 groups represented in the SDI-ILI dataset during the autumn 2009 A/H1N1pdm pandemic influenza outbreak. A box in row i and column j corresponds to the STE from group j to group i, where darker shades corresponds to higher STE. STE is calculated using the reduced four-symbol alphabet, obtained by collapsing symbols B and E as well as symbols C and D from the standard $m = 3$ symbolization.

12. Appendix

The relative reproduction matrix for the asymmetric transmission/uniform reporting rate simulations in Section 3.3 is

$$
\boldsymbol{r} = \begin{bmatrix}
1 & 1 & 2 & 2 & 2 & 1 & 1 & 1 & 1 & 1 & 1 & 1 \\
1 & 1 & 2 & 2 & 2 & 1 & 1 & 1 & 1 & 1 & 1 & 1 \\
1 & 1 & 4 & 4 & 4 & 1 & 1 & 1 & 1 & 1 & 1 & 1 \\
1 & 1 & 4 & 4 & 4 & 1 & 1 & 1 & 1 & 1 & 1 & 1 \\
1 & 1 & 4 & 4 & 4 & 1 & 1 & 1 & 1 & 1 & 1 & 1 \\
1 & 1 & 2 & 2 & 2 & 1 & 1 & 1 & 1 & 1 & 1 & 1 \\
1 & 1 & 2 & 2 & 2 & 1 & 1 & 1 & 1 & 1 & 1 & 1 \\
1 & 1 & 2 & 2 & 2 & 1 & 1 & 1 & 1 & 1 & 1 & 1 \\
1 & 1 & 2 & 2 & 2 & 1 & 1 & 1 & 1 & 1 & 1 & 1 \\
1 & 1 & 1 & 1 & 1 & 1 & 1 & 1 & 1 & 1 & 1 & 1 \\
1 & 1 & 1 & 1 & 1 & 1 & 1 & 1 & 1 & 1 & 1 & 1 \\
1 & 1 & 1 & 1 & 1 & 1 & 1 & 1 & 1 & 1 & 1 & 1
\end{bmatrix}. \tag{S49}
$$

The mean pairwise STE values for the asymmetric transmission/uniform reporting scenario depicted in Fig. 3A are

$$
\begin{bmatrix}
0 & 0.75 & 1.16 & 1.13 & 1.13 & 0.75 & 0.74 & 0.75 & 0.75 & 0.56 & 0.56 & 0.57 \\
0.77 & 0 & 1.15 & 1.14 & 1.14 & 0.75 & 0.75 & 0.74 & 0.74 & 0.56 & 0.55 & 0.56 \\
0.86 & 0.86 & 0 & 1.41 & 1.39 & 0.87 & 0.87 & 0.85 & 0.86 & 0.64 & 0.63 & 0.63 \\
0.85 & 0.85 & 1.38 & 0 & 1.39 & 0.85 & 0.86 & 0.87 & 0.86 & 0.63 & 0.63 & 0.63 \\
0.86 & 0.88 & 1.4 & 1.39 & 0 & 0.87 & 0.85 & 0.86 & 0.84 & 0.65 & 0.63 & 0.63 \\
0.73 & 0.74 & 1.15 & 1.16 & 1.15 & 0 & 0.75 & 0.73 & 0.72 & 0.56 & 0.55 & 0.57 \\
0.73 & 0.75 & 1.15 & 1.16 & 1.16 & 0.75 & 0 & 0.76 & 0.73 & 0.55 & 0.54 & 0.57 \\
0.74 & 0.75 & 1.14 & 1.15 & 1.14 & 0.74 & 0.76 & 0 & 0.74 & 0.55 & 0.55 & 0.54 \\
0.77 & 0.74 & 1.16 & 1.16 & 1.17 & 0.75 & 0.75 & 0.74 & 0 & 0.57 & 0.56 & 0.56 \\
0.62 & 0.63 & 0.91 & 0.9 & 0.91 & 0.64 & 0.63 & 0.63 & 0.62 & 0 & 0.5 & 0.49 \\
0.62 & 0.63 & 0.89 & 0.91 & 0.9 & 0.62 & 0.64 & 0.64 & 0.63 & 0.48 & 0 & 0.47 \\
0.61 & 0.62 & 0.9 & 0.89 & 0.9 & 0.62 & 0.61 & 0.61 & 0.62 & 0.48 & 0.48 & 0
\end{bmatrix} \times 10^{-2}. \tag{S50}
$$

The mean pairwise STE values for the uniform transmission/variable reporting scenario depicted in Fig. 3B are

$$\begin{bmatrix} 0 & 0.82 & 0.81 & 0.83 & 0.82 & 0.65 & 0.66 & 0.65 & 0.64 & 0.66 & 0.66 & 0.65 \\ 0.82 & 0 & 0.81 & 0.82 & 0.81 & 0.66 & 0.65 & 0.65 & 0.66 & 0.68 & 0.65 & 0.64 \\ 0.8 & 0.81 & 0 & 0.83 & 0.8 & 0.63 & 0.65 & 0.65 & 0.64 & 0.65 & 0.65 & 0.64 \\ 0.81 & 0.81 & 0.82 & 0 & 0.83 & 0.66 & 0.66 & 0.64 & 0.65 & 0.67 & 0.65 & 0.65 \\ 0.83 & 0.84 & 0.82 & 0.83 & 0 & 0.65 & 0.66 & 0.65 & 0.67 & 0.65 & 0.65 & 0.66 \\ 0.73 & 0.72 & 0.71 & 0.73 & 0.72 & 0 & 0.6 & 0.58 & 0.59 & 0.6 & 0.58 & 0.58 \\ 0.72 & 0.71 & 0.73 & 0.72 & 0.72 & 0.58 & 0 & 0.58 & 0.59 & 0.6 & 0.6 & 0.58 \\ 0.72 & 0.72 & 0.71 & 0.73 & 0.74 & 0.6 & 0.59 & 0 & 0.58 & 0.59 & 0.58 & 0.58 \\ 0.72 & 0.7 & 0.74 & 0.73 & 0.71 & 0.58 & 0.58 & 0.58 & 0 & 0.58 & 0.59 & 0.58 \\ 0.72 & 0.71 & 0.73 & 0.71 & 0.72 & 0.57 & 0.58 & 0.58 & 0.59 & 0 & 0.58 & 0.59 \\ 0.71 & 0.7 & 0.71 & 0.72 & 0.7 & 0.58 & 0.59 & 0.57 & 0.57 & 0.59 & 0 & 0.58 \\ 0.72 & 0.72 & 0.72 & 0.72 & 0.72 & 0.57 & 0.59 & 0.58 & 0.59 & 0.58 & 0.58 & 0 \end{bmatrix} \times 10^{-2}. \tag{S51}$$

The pairwise STE values for the autumn 2009 A/H1N1pdm pandemic influenza outbreak depicted in Fig. 4 are

$$\begin{bmatrix} 0 & 4.02 & 6.44 & 5.82 & 4.59 & 3.45 & 3.60 & 3.23 & 1.97 & 0.99 & 0.81 & 0.62 \\ 2.00 & 0 & 7.86 & 8.36 & 5.99 & 3.64 & 3.07 & 2.93 & 2.02 & 0.98 & 0.62 & 0.76 \\ 1.34 & 2.00 & 0 & 7.08 & 5.49 & 2.47 & 2.34 & 1.76 & 1.22 & 0.78 & 0.68 & 0.56 \\ 0.94 & 1.61 & 3.69 & 0 & 4.17 & 2.05 & 1.87 & 1.78 & 1.05 & 0.76 & 0.58 & 0.60 \\ 0.98 & 1.94 & 4.42 & 5.88 & 0 & 2.3 & 2.31 & 2.10 & 1.27 & 0.78 & 0.71 & 0.75 \\ 2.29 & 3.95 & 7.18 & 7.87 & 6.16 & 0 & 4.18 & 3.79 & 2.43 & 1.16 & 1.11 & 0.74 \\ 2.32 & 3.41 & 6.81 & 7.40 & 5.89 & 4.32 & 0 & 3.80 & 2.44 & 1.27 & 0.98 & 0.96 \\ 2.16 & 3.60 & 6.52 & 6.97 & 5.80 & 4.19 & 4.15 & 0 & 2.72 & 1.69 & 1.06 & 1.1 \\ 2.41 & 3.76 & 6.20 & 6.08 & 5.54 & 4.77 & 4.54 & 4.15 & 0 & 1.92 & 1.40 & 1.08 \\ 1.62 & 2.43 & 3.40 & 3.47 & 2.95 & 3.17 & 3.07 & 3.10 & 2.95 & 0 & 2.24 & 1.78 \\ 1.01 & 1.26 & 1.89 & 1.68 & 1.56 & 1.67 & 2.03 & 1.94 & 1.81 & 2.26 & 0 & 2.78 \\ 0.58 & 0.85 & 0.84 & 0.83 & 0.91 & 1.06 & 1.02 & 1.41 & 1.12 & 1.63 & 2.70 & 0 \end{bmatrix} \times 10^{-2}. \tag{S52}$$

The pairwise STE values for the autumn 2009 A/H1N1pdm pandemic influenza outbreak depicted in Fig. S19, using the collapsed four-symbol alphabet, are

$$\begin{bmatrix} 0 & 2.73 & 4.24 & 3.80 & 3.26 & 2.27 & 2.39 & 2.42 & 1.51 & 0.97 & 0.66 & 0.59 \\ 1.40 & 0 & 4.47 & 5.12 & 3.70 & 2.26 & 1.88 & 1.93 & 1.42 & 0.76 & 0.51 & 0.39 \\ 0.93 & 1.20 & 0 & 4.66 & 3.73 & 1.66 & 1.61 & 1.36 & 0.93 & 0.48 & 0.21 & 0.15 \\ 0.77 & 1.08 & 2.19 & 0 & 2.62 & 1.37 & 1.26 & 1.31 & 0.90 & 0.48 & 0.21 & 0.09 \\ 0.81 & 1.36 & 2.80 & 3.35 & 0 & 1.50 & 1.57 & 1.44 & 0.94 & 0.51 & 0.40 & 0.17 \\ 1.53 & 2.46 & 4.06 & 4.99 & 3.88 & 0 & 2.98 & 2.46 & 1.70 & 0.97 & 0.45 & 0.29 \\ 1.58 & 1.91 & 3.95 & 4.73 & 3.80 & 2.82 & 0 & 2.36 & 1.66 & 1.14 & 0.63 & 0.54 \\ 1.54 & 2.47 & 3.80 & 4.33 & 3.61 & 2.34 & 2.50 & 0 & 1.88 & 1.32 & 0.65 & 0.56 \\ 1.91 & 2.40 & 3.71 & 4.18 & 3.65 & 2.74 & 2.78 & 2.59 & 0 & 1.57 & 1.25 & 0.78 \\ 1.23 & 1.64 & 2.03 & 2.44 & 2.14 & 2.15 & 2.09 & 2.16 & 2.00 & 0 & 1.84 & 1.44 \\ 0.90 & 0.87 & 1.18 & 1.18 & 1.15 & 1.28 & 1.44 & 1.44 & 1.44 & 1.84 & 0 & 2.11 \\ 0.60 & 0.78 & 0.68 & 0.75 & 0.83 & 1.06 & 0.86 & 1.20 & 1.05 & 1.45 & 2.16 & 0 \end{bmatrix} \times 10^{-2}. \tag{S53}$$

References

[1] M. Staniek and K. Lehnertz, "Symbolic Transfer Entropy," *Phys. Rev. Lett.*, vol. 100, no. April, p. 158101, 2008.

[2] A. Kaiser and T. Schreiber, "Information transfer in continuous processes," *Physica D: Nonlinear Phenomena*, vol. 166, no. 1-2, pp. 43–62, 2002.

[3] T. Smieszek, M. Balmer, J. Hattendorf, K. W. Axhausen, J. Zinsstag, and R. W. Scholz, "Reconstructing the 2003/2004 H3N2 influenza epidemic in Switzerland with a spatially explicit, individual-based model," *BMC Infectious Diseases*, vol. 11, no. 1, p. 115, 2011.

[4] M. A. Jhung, D. Swerdlow, S. J. Olsen, D. Jernigan, M. Biggerstaff, L. Kamimoto, K. Kniss, C. Reed, A. Fry, L. Brammer, J. Gindler, W. J. Gregg, J. Bresee, and L. Finelli, "Epidemiology of 2009 pandemic influenza a (H1N1) in the United States," *Clinical Infectious Diseases*, vol. 52, no. SUPPL. 1, pp. 13–26, 2011.

[5] W. Yang, M. Lipsitch, and J. Shaman, "Inference of seasonal and pandemic influenza transmission dynamics," *Proceedings of the National Academy of Sciences*, vol. 112, no. 9, p. 201415012, 2015.

[6] S. Ghebrehewet, P. MacPherson, and A. Ho, "Influenza," *BMJ*, vol. 6258, p. i6258, dec 2016.

[7] J. R. Gog, S. Ballesteros, C. Viboud, L. Simonsen, O. N. Bjørnstad, J. Shaman, D. L. Chao, F. Khan, and B. T. Grenfell, "Spatial Transmission of 2009 Pandemic Influenza in the US," *PLoS Computational Biology*, vol. 10, p. e1003635, jun 2014.

[8] M. Biggerstaff, M. Jhung, L. Kamimoto, L. Balluz, and L. Finelli, "Self-reported influenza-like illness and receipt of influenza antiviral drugs during the 2009 pandemic, United States, 2009-2010," *American Journal of Public Health*, vol. 102, no. 10, pp. 2009–2010, 2012.
